# Proteomic Analysis of 2,4,6-Trinitrotoluene Degrading Yeast *Yarrowia lipolytica*

**DOI:** 10.3389/fmicb.2017.02600

**Published:** 2017-12-22

**Authors:** Irina V. Khilyas, Guenter Lochnit, Olga N. Ilinskaya

**Affiliations:** ^1^Institute of Fundamental Medicine and Biology, Kazan (Volga Region) Federal University, Kazan, Russia; ^2^Protein Analytics, Institute of Biochemistry, Faculty of Medicine, Justus Liebig University Giessen, Giessen, Germany

**Keywords:** 2,4,6-trinitrotoluene, TNT, biodegradation, *Yarrowia lipolytica*, proteomic assay, old yellow enzymes

## Abstract

2,4,6-trinitrotoluene (TNT) is a common component of many explosives. The overproduction and extensive usage of TNT significantly contaminates the environment. TNT accumulates in soils and aquatic ecosystems and can primarily be destroyed by microorganisms. Current work is devoted to investigation of *Yarrowia lipolytica* proteins responsible for TNT transformation through the pathway leading to protonated Meisenheimer complexes and nitrite release. Here, we identified a unique set of upregulated membrane and cytosolic proteins of *Y. lipolytica*, which biosynthesis increased during TNT transformation through TNT-monohydride-Meisenheimer complexes in the first step of TNT degradation, through TNT-dihydride-Meisenheimer complexes in the second step, and the aromatic ring denitration and degradation in the last step. We established that the production of oxidoreductases, namely, NADH flavin oxidoreductases and NAD(P)+-dependent aldehyde dehydrogenases, as well as transferases was enhanced at all stages of the TNT transformation by *Y. lipolytica*. The up-regulation of several stress response proteins (superoxide dismutase, catalase, glutathione peroxidase, and glutathione *S*-transferase) was also detected. The involvement of intracellular nitric oxide dioxygenase in NO formation during nitrite oxidation was shown. Our results present at the first time the full proteome analysis of *Y. lipolytica* yeast, destructor of TNT.

## Introduction

Nitroaromatic compounds play an important role in the synthesis of explosives, pharmaceutical compounds, pesticides and herbicides (Boelsterli et al., 2006). Of all nitroaromatic compounds, explosives are of particular concern to the environment (Symons and Bruce, 2006). Military products and waste containing explosives are regularly dumped on land and into water in the process of storage, dismantling, or destruction (Letzel et al., 2003). 2,4,6-trinitrotoluene (TNT) is a common component of many explosives. The overproduction and extensive usage of TNT significantly contaminates the environment. TNT accumulates in soils and aquatic ecosystems and can primarily be destroyed by microorganisms (Mulla et al., 2014). The reactive nitro groups located on an electron-deficient aromatic ring possess electron-attractive potential that can influence the stability of TNT. The TNT transformation mechanism involves a two-electron reduction of nitro groups which leads to the formation of hydroxylamino- (HADNTs) and amino-derivatives (ADNTs) by nitroreductases under aerobic conditions (Caballero et al., 2005). The resulting HADNTs and ADNTs derivatives possess some negative properties, such as high toxicity, mutagenicity, and carcinogenicity (Spanggord et al., 1995).

The microbial degradation of TNT through the formation of TNT-Meisenheimer complexes is a unique path leading to the release of nitrogroups and conditioning the enzymatic cleavage of the aromatic ring, which is followed by its complete degradation (Ziganshin and Gerlach, 2014). *Enterobacter cloacae* PB2, *Pseudomonas fluorescence* I-C, and *Pseudomonas putida* JLR11 are the aerobic bacteria that hydrogenate the TNT aromatic ring and trigger the release of ${NO}_{2}^{-}$ by the family of old yellow enzymes (OYEs) (Pak et al., 2000; Wittich et al., 2008). OYEs and their homologs can be found in prokaryotic and eukaryotic organisms (Singh, 2014). Previously, the OYE homologs were detected in higher plants (Strassner et al., 1999). Specifically, TNT was found to activate the process of nitroreductases in the root cell cytosol of soybean, which reduced the nitro group of the obtained TNT products (Adamia et al., 2006). It was established that the detoxification strategy of plants confined to the conjugation and compartmentalization of xenobiotic compounds in the vacuole or cell wall, resulting in the formation of non-extractable compounds (Sens et al., 1999). However, no direct evidence for OYEs participation in the TNT transformation by micromycetes has been provided so far. Although the OYEs homologs can be found in different species of fungi, only *Irpex lacteus* accumulates TNT-hydride-Meisenheimer complexes (Nizam et al., 2014). It is known that OYE members are involved in the oxidative stress response (Farah and Amberg, 2007). A strong correlation between the participation of OYEs homologs in the transformation of nitroaromatic compounds and detoxification of reactive oxygen species (ROS) was reported in previous studies (Fitzpatrick et al., 2003). In particular, *Bacillus subtilis* protein (YqjM) related to OYE homologs was found to reduce nitroesters and nitroaromatic compounds and to be induced by hydrogen peroxide (Fitzpatrick et al., 2003).

The study of yeast proteins that trigger the transformation of nitroaromatic compounds contributes to a better understanding of a wide range of processes attributed to biotransformation of pharmacological, agricultural and industrial toxic compounds. In this paper, we present the results of an investigation into the proteomic profiling of TNT-degrading aerobic yeast *Yarrowia lipolytica* VKPM Y-3492. In our study, we identified a unique set of upregulated membrane and cytosolic proteins of *Y. lipolytica*, which biosynthesis increased during TNT transformation through TNT-monohydride-Meisenheimer complexes in the first step of TNT degradation, through TNT-dihydride-Meisenheimer complexes in the second step, and the aromatic ring denitration and degradation in the last step. We established that the production of oxidoreductases, namely, NADH flavin oxidoreductases and NAD(P)+-dependent aldehyde dehydrogenases, as well as transferases was enhanced at all stages of the TNT transformation by *Y. lipolytica*. Simultaneously, we detected the upregulation of several stress response proteins [superoxide dismutase (SOD), catalase, glutathione peroxidase, and glutathione *S*-transferase (GST)]. Finally, we established upregulation of yeast intracellular nitric oxide dioxygenase (NOD) participating in NO release. Finally, we established the involvement of intracellular NOD in NO formation during nitrite oxidation. This finding supports the biogenic path of NO formation in addition to abiotic generation illustrated in previous studies (Ziganshin et al., 2010; Khilyas et al., 2013).

## Materials and Methods

### Chemicals

TNT (purity, 99%) was purchased from ChemService (West Chester, PA, United States).

### Yeast Strain and Culture Conditions

*Yarrowia lipolytica* was grown aerobically at 30°C for 1 day on Sabouraud agar medium containing (per liter) glucose 10 g, peptone 10 g, yeast extract 5 g, NaCl 0.25 g, and agar 20 g. Yeast cells were harvested, washed with 16 mM phosphate buffer (pH 7.0) and added into 250 mL Erlenmeyer flasks containing 50 mL of synthetic medium. The synthetic medium was composed of 28 mM glucose, 7.6 mM (NH_4_)_2_SO_4_, 2 mM MgSO_4_ and buffered with 16 mM K-Na-phosphate buffer to pH 7.0. The initial cell concentration was adjusted to an optical density (600 nm, A_600_) of 1.0, and growth was measured using a Lambda 35 spectrophotometer (Perkin Elmer) with cell-free (filtered) culture medium as reference. TNT was added as ethanol stock solution to a final concentration 440 μM, and flasks were incubated at 30°C with shaking speed 150 rpm. TNT-free control experiments contained pure ethanol (1.33 mL absolute ethanol into 50 mL medium). Samples of yeast cells were taken during culture growth at time points corresponding maximum formation of analytically measured TNT metabolites. After centrifugation, yeast biomass was washed with 16 mM phosphate buffer twice, frozen in liquid nitrogen, lyophilized, and stored at -80°C. These samples were used for proteins analysis. All experiments were set up in two biological repetitions. To exclude proteins of the first step of TNT transformation, Boolean algorithm for binary data sets of TNT-treated and TNT-untreated yeast proteins at pH 4.6 and pH 6.6 was applied.

### Analytical Methods

TNT and its biotransformation products were detected with a HPLC (Thermo Scientific^TM^ Dionex^TM^ UltiMate^TM^ 3000) equipped with autoinjector, diode array detector, the column oven, and Supelcosil (C-8) column (150 by 4.6 mm; particle size, 5 μm), as described previously (Borch and Gerlach, 2004; Khilyas et al., 2013). Nitrates and nitrites were detected using ion chromatograph 761 Compact IC (Metrohm, Sweden) equipped with a Metrosep A SUPP 5 column (150/4.0; particle size, 5 μm).

### Proteome Analysis

TE-buffer (EDTA, pH 8.0, Tris base) containing 1% Protease Inhibitor Mix HP (Serva, Heidelberg, Germany) was added to the yeas biomass. Yeast cells were disrupted by glass beads using a Potter-Elvehejem homogenizer at 4°C (Wheaton, Millville, NJ, United States). After centrifugation (14000 *g* for 30 min) supernatant containing cytoplasmic proteins was collected. The pellet including membrane-associated proteins was resuspended in an ice-cold solution (8 M urea and 2 M thiourea). Delipidation of supernatant and pellet was performed by methanol-chloroform method (Wessel and Flügge, 1984). Protein quantification was carried out by 2-D Quant kit (GE Healthcare, United States). Cytosolic or membrane-associated proteins (150 μg) were subjected to gel strips (pH 7–11 NL, 13 cm) in sample buffer [4% CHAPS, 30 mM DTT, 20 mM Tris-base, 2% IPG buffer, 1% bromophenol blue (added immediately before use)] and maintained 24 h at room temperature for rehydration. Then, strips were placed in a horizontal running tray for IEF and covered with mineral oil to prevent dehydration during electrophoresis (Wenge et al., 2008). Electrophoresis was performed at 0–100 V (1 mA) for 5 h, 100–3500 V (1 mA) for 6 h and 3500 V (1 mA) for 6 h at 20°C. Strips were washed with 1% DTT for 15 min, with 4% iodoacetamide for 15 min and three times with 50 mM Tris buffer (pH 6.8). Equilibrated strips were transferred on 12.5% polyacrylamide SDS gel (18 cm × 20 cm) using tweezers. Electrophoresis conditions per one gel were 15 mA (600 V, 50 W) for 15 min and 110 mA (600 V, 50 W) for 6 h. Densitometric analyses were done with the PDQuest software (Bio-Rad)after Coomassie-staining.

### Tryptic in-Gel Digestion of Proteins

Coomassie-stained protein spots (*n* = 404) were excised with the ExQuest Spot Cutter (BioRad) and transferred into 96-wells plates. Digestion of protein was done using liquid handling system (Microstarlet, Hamiltonrobotics, Martinsried, Germany). Gel plugs were washed 10 min with 150 μl 50% acetonitrile (ACN), dehydrated 2 min with 150 μl 100% ACN, swelled 5 min with 150 μl 50 mM NH_4_HCO_3_, dehydrated 2 min with 150 μl 100% ACN. The proteins were digested for 120 min at 45°C in 25 mM NH_4_HCO_3_ containing 0.5 mg trypsin (sequencing grade, Promega, Mannheim, Germany). Peptides were stabilized by 10 μL 0.1% TFA and stored at 21°C until use (Wenge et al., 2008).

### MALDI-TOF MS

MALDI-TOF MS was performed using an UltraflexI TOF/TOF mass spectrometer (Bruker Daltonics, Bremen, Germany) equipped with a nitrogen laser and a LIFT-MS/MS facility. The instrument was operated in the positive-ion reflectron mode using 2,5-dihydroxybenzoic acid (Sigma) in 50% ACN/1% phosphoric acid as matrix. Acquired sum spectra consist of 200–400 single spectra. For data processing and instrument control the Compass 1.1 software package consisting of FlexControl 2.4, FlexAnalysis 2.4, and ProteinScape 3.0 was used.

### Database Search

Proteins were identified by MASCOT peptide mass-fingerprint search^1^ using the MSDB database. The search was restricted to *Yarrowia* with a mass tolerance of 100 ppm and carbamidomethylation of cysteine as global modification and oxidation of methionine as variable modification.

## Results

### Three Steps of TNT Transformation by *Y. lipolytica*

After 3 h of cultivation in TNT-free medium, yeast cells were grown to 1.7 units (cell density of 600 nm), whereas in TNT medium cells reached the density of 1.3 units after 5 h of cultivation only (Supplementary Figure S1A). In view of the fact that the biosynthesis for a spectrum of synthesized proteins depends on pH, we sampled the yeast biomass at different time points (3 and 5 h), but at equal levels of pH = 6.5. HPLC data showed that the TNT transformation through aromatic ring reduction led to a maximum accumulation of TNT-monohydride complexes (H^-^-TNT) after 5 h of yeast cultivation. The maximum concentrations 3-H^-^-TNT and 1-H^-^-TNT were 289 and 17 μM, accordingly (Supplementary Figure S1B). At the same time, the TNT transformation via nitro group reduction resulted in the accumulation of 26 μM of 2-hydroxylamino-4,6-dinitrotoluene (2-HADNT), 46 μM of 4-hydroxylamino-2,6-dinitrotoluene (4-HADNT) and 43 μM of nitrites (Supplementary Figure S1C). Thus, the yeast cells grown for 3 h without TNT and the cells grown for 5 h with TNT were used for protein analysis. We relate these time point to the first step of TNT transformation, which leads to the accumulation of TNT-monohydride complexes.

After 12 h of incubation, the pH of culture medium decreased to 4.6. Under these conditions, the TNT-monohydride complexes transformed into TNT-dihydride complexes (3,5-2H^-^-TNT⋅H^+^), and the concentration of 2-HADNT and 4-HADNT increased to 49 and 96 μM, respectively. The appearance of 2,4-DNT (11 μM) during 3,5-2H^-^-TNT⋅H^+^ destruction was associated with a release of 133 μM ${NO}_{2}^{-}$, which was partially oxidized to ${NO}_{3}^{-}$ and accompanied by NO formation as it was shown previously (Ziganshin et al., 2010; Khilyas et al., 2013). The time-point of 12 h was chosen for the second step of protein analysis.

The continued cultivation of *Y. lipolytica* for 24 h resulted in a pH shift to a strong acidic range (pH 3.1-3.6) and the intensive destruction of TNT-mono- and dihydride complexes. However, HADNTs, 4-ADNT, and 2,4-DNT, as well as ${NO}_{3}^{-}$. As a product of ${NO}_{2}^{-}$ oxidation could be still found in growth medium (Supplementary Figures S1B,C). This time point (24 h) was chosen for the last step of protein analysis.

### Twenty Proteins of *Y. lipolytica* Are Up-Regulated at Maximal TNT-Monohydride Complexes Formation

Next, the membrane and cytosolic proteins were analyzed to determine the quantitative and qualitative changes in the protein profiling of *Y. lipolytica* based on the maximum accumulation of TNT-monohydride complexes after 5 h of cultivation. Approximately, 1300–2000 protein spots were detected on each gel: eight gels correspond to TNT-free cultivation (four for membrane proteins and four for cytosolic proteins) and eight gels were obtained based on the cultivation with TNT. For twenty revealed protein spots, the difference (*p* < 0.05) between TNT-treated and TNT-untreated yeast was more than fivefold. All these proteins were up-regulated in the presence of TNT. The influence of pH alteration in medium was excluded (Supplementary Table S1).

**FIGURE 1:**
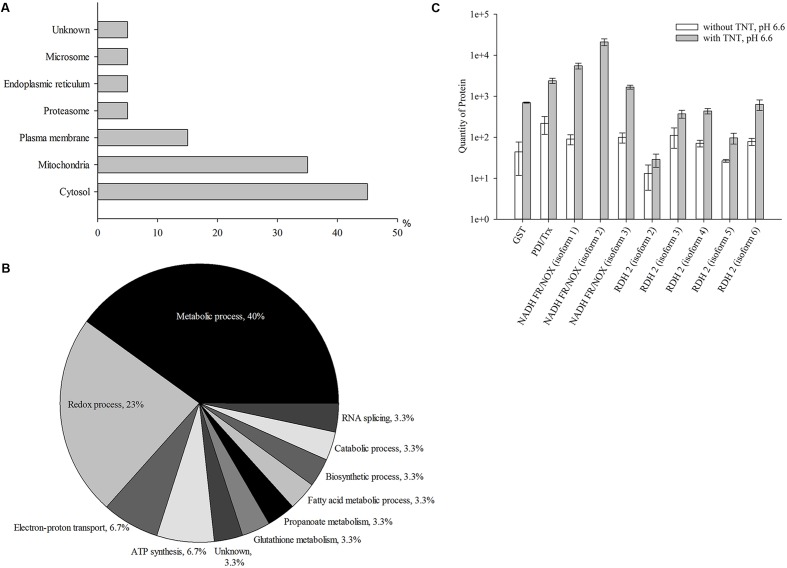
Up-regulated proteins at the first stage of TNT transformation by *Yarrowia lipolytica*: their intracellular localization **(A)**; participation in metabolic processes **(B)** and the quantity of major proteins maximal up-regulated by TNT **(C)**. **(A,B)** For 100% all identified proteins at the first stage of TNT transformation were taken. **(C)** Error bars represent the standard deviation of duplicate biological experiments with two technical repetitions. GST, glutathione *S-*transferase; PDI/Trx, protein disulfide isomerase/thioredoxin; NADH FR/NOX, NADH flavin oxidoreductases/NADH oxidases; RDH, retinal dehydrogenase 2.

**Figure 1A** shows the intracellular distribution of 20 up-regulated proteins in the compartments of yeasts and their role in the cellular processes. A significant number of the up-regulated proteins could be assigned to cytosolic (45% of the total proteins) and mitochondrial (35% of the total proteins) compartments of yeast cells; 15% of all up-regulated proteins were related to plasma membrane proteins. Five percent of other proteins were associated with proteasomes, endoplasmic reticulum, and microsomes, respectively. About 5% of proteins could not be identified. More detailed information is presented in Supplementary Table S1. The cytosolic and membrane proteins from *Y. lipolytica* were involved in the metabolic process (40%), redox process (23%), electron-proton transport (7%), ATP synthesis (7%), biosynthetic process (4%), catabolic process (4%), fatty acid metabolic process (3%), RNA splicing (3%), propanoate metabolism (3%), and glutathione metabolism (3%). The functions performed by 3% of proteins remained unknown (**Figure 1B**).

In general, TNT primarily induced the biosynthesis of oxidoreductases and transferases of *Y. lipolytica*. Three membrane isoforms of NADH flavin oxidoreductases/NADH oxidases demonstrated a higher up-regulation (by 16–60 times) (**Figure 1C**). Five different isoforms of retinal dehydrogenases 2, belonging to the NAD(P)+-dependent aldehyde dehydrogenase superfamily (ALDH-SF), also increased by eight times. GST and disulfide isomerase/thioredoxin showed a 15- and 10-fold increase, respectively (**Figure 1C**). Thus, the first step of TNT transformation was associated with an increased level of yeast proteins catalyzing metabolic redox reactions.

### Proteins of *Y. lipolytica* Are Up-Regulated at Maximal TNT-Dihydride Complexes Formation

**FIGURE 2:**
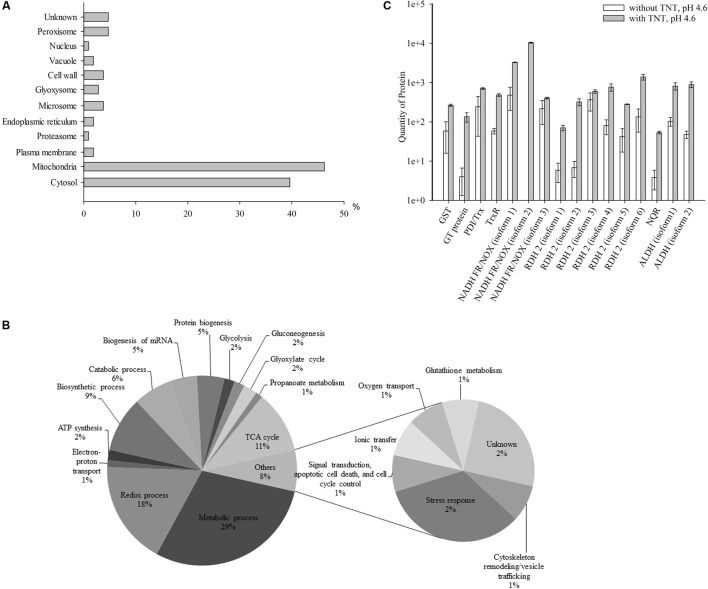
Up-regulated proteins at the second stage of TNT transformation by *Y. lipolytica*: their intracellular localization **(A)**; participation in metabolic processes **(B)** and the quantity of major proteins maximal up-regulated by TNT transformation products. **(A,B)** For 100% all identified proteins at the second stage of TNT transformation were taken. **(C)** Error bars represent the standard deviation of duplicate biological experiments with two technical repetitions. GST, glutathione *S*-transferase; GT, glutathione transferase protein; PDI/Trx, protein disulfide isomerase/thioredoxin; TrxR, thioredoxin reductase; NADH FR/NOX, NADH flavin oxidoreductases/NADH oxidases; RDH, retinal dehydrogenase 2; NQR, NADPH:quinone oxidoreductase; ALDH, aldehyde dehydrogenases.

The second step of TNT transformation was characterized by the accumulation of TNT-dihydride complexes. In total, 102 up-regulated proteins in cytosolic and membrane fractions were revealed, that is, about five times higher compared with the first step of transformation (Supplementary Table S2). In addition, the number of mitochondrial proteins grew from 35 to 48% compared with the first step. At the same time, the number of cytosolic proteins insignificantly reduced (41.2%) (**Figures 1A**, **2A**). Others proteins were localized in peroxisomes (4.9%), microsomes and cell wall (3.9% each), plasma membrane, endoplasmic reticulum and vacuoles (2% of each), proteasomes and nucleus (1% of each), while the localization of 4.9% of the proteins remained unknown (**Figure 2A**).

**Figure 2B** illustrates the functional classification of up-regulated intracellular *Y. lipolytica* proteins which can be attributed to the accumulation of TNT-dihydride complexes at pH 4.6. The identified proteins participated in metabolic (29%) and redox (17%) processes, and the tricarboxylic acid (TCA) cycle (10%), as well as in biosynthetic (9%) and catabolic (6%) processes (**Figure 2B** and Supplementary Table S2). In the second step, we also obtained the insignificant amount of proteins that were not up-regulated during the first stage of TNT transformation. These proteins participated in protein biogenesis (5%), biogenesis of RNA (5%), ATP synthesis (2%), glycolysis (2%), gluconeogenesis (2%), glyoxylate cycle (2%), propanoate metabolism (1%), electron-proton transport (1%), and other processes (8%) (**Figure 2B** and Supplementary Table S2).

Interestingly, the level of NADH flavin oxidoreductases/NADH oxidases was much lower than in the first step of TNT transformation (corresponding to a sixfold increase only) (**Figure 2C**). Six isoforms of retinal dehydrogenase 2 were up-regulated 45 times. At this stage only, two isoforms of acetaldehyde dehydrogenases were up-regulated. The isoform 2 of NADH flavin oxidoreductase was not detected in the TNT-free proteome at both stages of TNT transformation, whereas the NADPH:quinone reductase was for the first time detected at the second stage (**Figure 2C**). The GST and disulfide isomerase/thioredoxin were up-regulated, although the level of up-regulation was lower compared with that achieved in the first step of TNT transformation (by 4.5 times only) (**Figure 2C**). Additionally, the level of glutathione transferase protein showed a 33-fold increase (**Figure 2C**). Finally, we established an eightfold up-regulation of thioredoxin reductase (**Figure 2C**).

After 12 h of TNT transformation, we observed an increase in the level of proteins involved in the regulation of RNA biogenesis (transcription and translation elongation) and protein biogenesis (protein folding, modification, and proteolysis) (Supplementary Table S2). Several TNT-induced heat shock proteins (HSP), including HSP 60 (GroEL), HSP78 and piso0_004415 (ATPases associated with a wide variety of cellular activities), ATP-dependent molecular chaperone HSC82, and chaperone protein (DnaK) were identified (Supplementary Table S2). Furthermore, saccharopepsin and carboxypeptidase C, which have a specific location in vacuoles and perform multiple beneficial functions inside the yeast cells, such as proteolysis, were identified (Supplementary Table S2).

The amount of unknown and hypothetical proteins activated at the second stage of TNT-transformation was significantly increased compared with the stage of accumulating the TNT-monohydride complexes (Supplementary Tables S1, S2). Thus, the second step of TNT transformation was characterized by the sustained increase in yeast proteins participating in metabolic redox reactions, proteolysis, and stress-response.

### Proteins of *Y. lipolytica* Are Up-Regulated at Stage of TNT-Hydride Complexes Destruction

**FIGURE 3:**
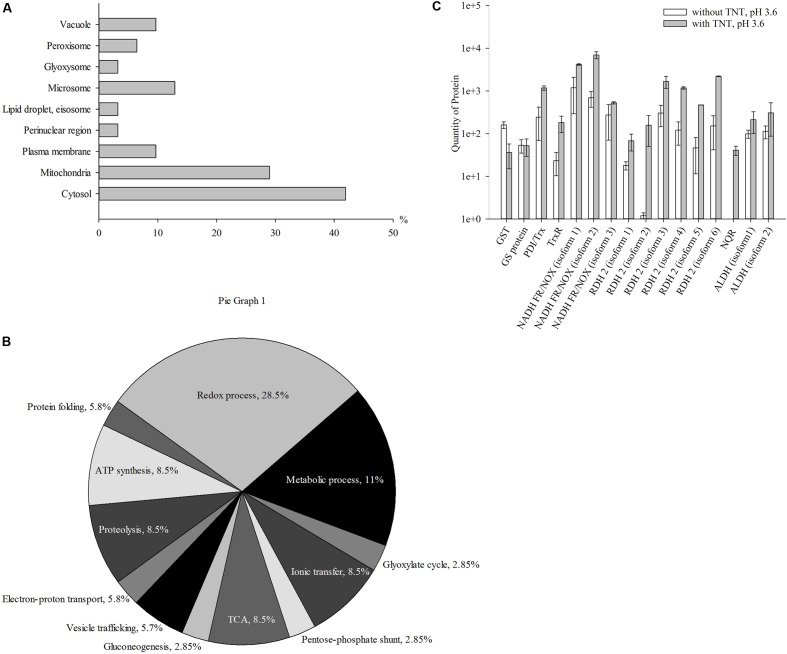
Up-regulated proteins at the third stage of TNT transformation by *Y. lipolytica*: their intracellular localization **(A)**; participation in metabolic processes **(B)** and the quantity of major proteins maximal up-regulated by TNT transformation products. **(A,B)** For 100% all identified proteins at the third stage of TNT transformation were taken. **(C)** Error bars represent the standard deviation of duplicate biological experiments with two technical repetitions. GST, glutathione *S*-transferase; GT, glutathione transferase protein; PDI/Trx, protein disulfide isomerase/thioredoxin; NADH FR/NOX, NADH flavin oxidoreductases/NADH oxidases; RDH, retinal dehydrogenase 2; ALDH, aldehyde dehydrogenases.

The third step of TNT transformation was characterized by the destruction of TNT-dihydride complexes. Specifically, we identified 31 up-regulated proteins in the cytosolic and membrane fractions. **Figure 3A** shows the intracellular distribution of up-regulated proteins. A significant number of the up-regulated proteins were localized in the cytosolic (41.9%) and mitochondrial (29%) compartments of yeast cells (**Figure 3A**). Other proteins were localized in microsomes (12.9%), plasma membrane (9.7%) and vacuoles (9.7%), peroxisome (6.5%), the perinuclear region (3.2%), lipid droplet (3.2%), eisosomes (3.2%) and glyoxysomes (3.2%) (**Figure 3A**).

**Figure 3B** illustrates the functional classification of up-regulated intracellular *Y. lipolytica* proteins that can be attributed to the destruction of TNT-dihydride complexes at pH 3.6. The identified proteins participated in metabolic (11%) and redox (29%) processes, TCA cycle, ATP synthesis, ionic transfer and proteolysis (8.6% of each), vesicle trafficking (5.7%) and cell wall biogenesis (5.7%) (**Figure 3B** and Supplementary Table S3).

The last stage of TNT transformation by *Y. lipolytica* led to the inhibition of metabolic processes and the activation of redox processes (**Figure 3B**). The average amount of proteins participating in energetic processes insignificantly decreased compared with the first and the second stages of TNT transformation, although the part of cellular processes occurring in plasma membrane, microsomes and vacuoles was higher (**Figures 1A**, **2A**, **3A**).

**Figure 3C** illustrates the induction of six retinal dehydrogenases 2, NADPH quinone reductase, and two isoforms of the NAD(P)+-dependent aldehyde dehydrogenase. The isoform 2 of retinal dehydrogenase 2 particularly increased in the third step of TNT transformation.

Furthermore, an increase in the level of sphingolipid long chain base-responsive protein located in eisosome nearly to plasma membrane was detected (Supplementary Table S3). In addition, we observed the overexpression of the potassium channel subunit, which was involved in secretory and endocytic vesicular trafficking pathways. Among HSPs, only HSP 60, or GroEL was found to be up-regulated (Supplementary Table S3).

The yeast cells that adapted to the TNT-induced stress reached an optical density similar to the one that could be observed for the TNT-free system at the end of the third stage of TNT transformation (Supplementary Figure S1A). Yeast cells performed the destruction of TNT-dihydride complexes by NADH flavin oxidoreductases/NADH oxidases, whereas a decrease in HADNTs and ADNTs was entailed by proteins of the NAD(P)+-dependent aldehyde dehydrogenase superfamily (ALDH-SF) (**Figures 1C**, **2C**, **3C**). Three isoforms of NADH flavin oxidoreductases/NADH oxidases, as well as disulfide isomerase/thioredoxin, thioredoxin reductase, and peroxiredoxin were found to be up-regulated at all stages of TNT transformation (**Figures 1C**, **2C**, **3C**). Generally, the predominant part of the up-regulated enzymes belonged to oxidoreductases. This fact points to the role of such enzymes in TNT transformation.

### Identification of Proteins Involved in Detoxification Processes of TNT Biotransformation

**FIGURE 4:**
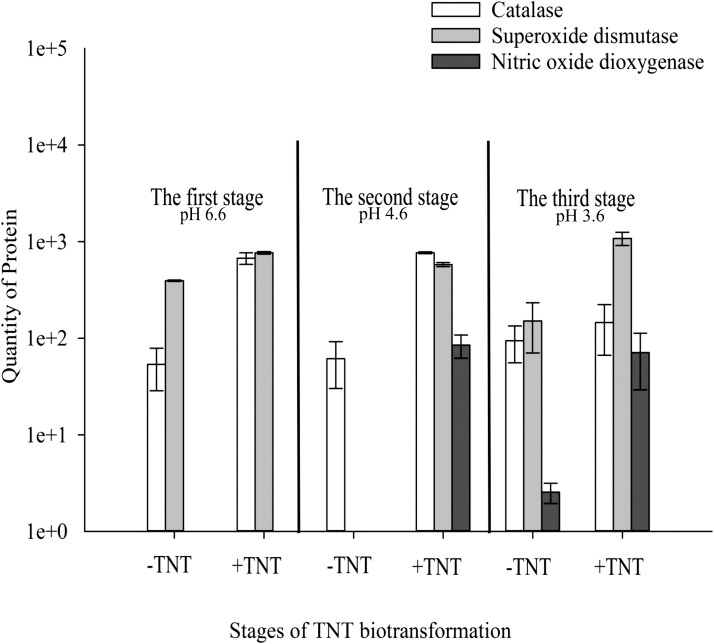
Quantitative changes of up-regulated intracellular proteins of *Y. lipolytica* involved in detoxification of reactive oxygen and nitrogen species during all stages of TNT biotransformation. Error bars represent the standard deviation of duplicate biological experiments with two technical repetitions.

A significant part of the up-regulated enzymes besides NADH flavin oxidoreductases/NADH oxidases was related to transferases (**Figure 4**). An increase in the level of these enzymes reflects the activation of the second phase of xenobiotics detoxification. Active in the first phase of TNT transformation, oxidoreductases led to the formation of TNT metabolites, namely, HADNTs and ADNTs, which could not be fully utilized by *Y. lipolytica* (Ziganshin et al., 2007). Therefore, the conjugation of these compounds with cellular substrates such as glutathione was necessary to excrete the toxic substances. Indeed, we could observe a high level of transferases during the entire cycle of TNT transformation (**Figure 4**).

The TNT transformation process through the aromatic ring reduction and reduction of nitro groups by *Y. lipolytica* was closely associated with the generation of ROS. Catalase and SOD could be found at the up-regulated level during all stages of TNT transformation (**Figure 4**). It is important to note that the high level of catalase and SOD was observed in TNT-untreated cells, which is characteristic of yeast cell growth under aerobic conditions.

Previously, we demonstrated the extracellular generation of nitric oxide (NO) during TNT transformation by *Y. lipolytica* using ESR spectroscopy (Khilyas et al., 2013). ESR spectra confirmed that ${NO}_{2}^{-}$ was converted into NO and ${NO}_{3}^{-}$ following a decrease in pH below 4.5, which coincides with the biotransformation of 3H^-^-TNT to 3,5-2H^-^-TNT⋅H^+^ (Khilyas et al., 2013). In this study, we observed a unique induction of intracellular NOD of *Y. lipolytica* at the second and third stage of TNT transformation (**Figure 4**). NOD catalyzed the reaction of superoxide anion detoxification with the production of less toxic ${NO}_{3}^{-}$ and was induced simultaneously to NO generation. However, minor amounts of NOD were detected in the TNT-untreated cells when pH of the medium reduced to 3.6 (**Figure 4**). The cellular generation of NO in the absence of TNT could be associated with NO-synthase activity which catalyzed the reaction between L-arginine, molecular oxygen and NAD(P)H (Xia et al., 2000; Ignarro et al., 2002).

## Discussion

TNT is a nitroaromatic agent which is highly adapted for military needs as it can spread and persist in soils, surface, and groundwater (Rylott et al., 2011). Members of the protein families involved in TNT biotransformation are quite known and well-characterized. Microorganisms use several metabolic pathways of TNT transformation. Aerobic bacteria initiate HADNTs and ADNTs formation by nitroreductases and PETN reductase, although PETN reductase, XenB reductase and members of the OYE family participate as well in the production of a Meisenheimer complex with following TNT denitration (French et al., 1998; Pak et al., 2000; Oh et al., 2001; Khan et al., 2002; Caballero et al., 2005; Iman et al., 2017). We confirm by proteomic analysis the upregulation of several proteins in *Y. lipolytica* treated by TNT. Three steps of TNT transformation by yeasts led to upregulation of NADH flavin oxidoreductases/NADH oxidases isoforms. Protein-disulfide isomerase was detected not only in yeast TNT-treated proteome, but also in *Stenotrophomonas maltophilia* (Lee et al., 2009). It could be noted, that the part of expressed proteins has not any relation to degradation enzymes. The pool of heat-shock proteins of *Y. lipolytica* (see Supplementary Material), *Stenotrophomonas* sp. (Ho et al., 2004), *Pseudomonas* sp. (Lee et al., 2008) and alginate-producing enzymes of *Pseudomonas* sp. growing on the reach medium with TNT (Cho et al., 2009) has been detected. However, no comprehensive metaproteome analysis of non-conventional yeasts based on an in-depth investigation of key enzymes which are responsible for the formation of TNT metabolite has been conducted to date.

Pathways of nitroaromatic compound biotransformation through enzymatic oxidative and reductive reactions are widely distributed among aerobic and anaerobic bacteria, fungi, plants, animals, and humans (Williams and Bruce, 2002; Stenuit and Agathos, 2010; Claus, 2014). The oxygenolytic mechanisms of TNT transformation could not be observed in living organisms due the chemical structure of such molecules (Esteve-Nunez et al., 2001). Hence, the microorganisms used different reduction pathways of TNT biotransformation preferentially leading to hydroxylamino and amino metabolites (Williams et al., 2004). Several strains carried out the reduction of aromatic ring with the formation of hydride Meisenheimer complexes, whereas some groups harbored both pathways (Smets et al., 2007).

**FIGURE 5:**
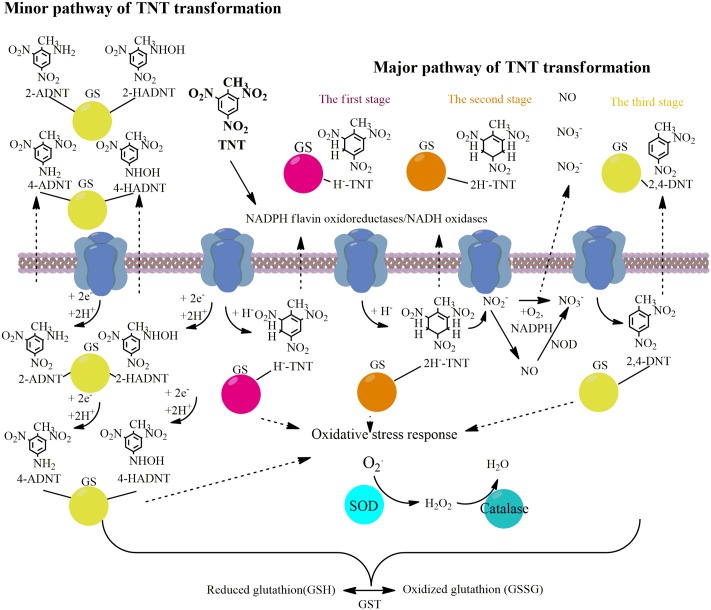
Schema illustrating the main pathways and key upregulated enzymes of TNT transformation by *Y. lipolytica*.

Earlier, it was shown that *Y. lipolytica* formed TNT hydride-Meisenheimer complexes during the first 6 h of cultivation (Zaripov et al., 2004; Ziganshin et al., 2007). This points to the fact that the most energetic equivalents are used in reductive reactions of yeast in the beginning of its growth. Thus, the enzymes induced by TNT in the initial step of transformation participate in energy-dependent processes. Furthermore, we identified the upregulation of NADH flavin oxidoreductases/NADH oxidases and GST (**Figures 1–3**). Oxidoreductases localized in the plasma membrane of yeast cells are the pioneering proteins in TNT transformation via TNT monohydride-Meisenheimer complexes (**Figures 1**–**3**, **5** and Supplementary Table S1). Different molecular masses of NADH flavin oxidoreductases/NADH oxidases indicate that the enzyme exists in several isoforms (Supplementary Table S1). The 45% similarity of NADH flavin oxidoreductases/NADH oxidases with PETN reductase of *E. cloacae* PB2 suggests a reaction mechanism via TNT-hydride complexes formation by *Y. lipolytica* occurs by the transfer of hydride from the enzyme to the aromatic ring (French et al., 1998; Khan et al., 2002).

Further, the spectrum of up-regulated oxidoreductases expanded during the formation of dihydride-Meisenheimer complexes. Similarly to retinal dehydrogenase 2, acetaldehyde dehydrogenases participated in 2e^-^ reductions processes. NADPH quinone reductase catalyzing the two-electron reduction of quinones and nitroaromatic compounds is up-regulated at the stage of maximum accumulation of TNT-dihydride complexes (**Figures 2C**, **5**). In addition, the second stage of TNT transformation was characterized by the up-regulation of glutathione transferase protein (**Figures 2C**, **5**).

The complete destruction of TNT-dihydride complexes occurred in the stationary phase of *Y. lipolytica* growth, which corresponds to the third stage of TNT transformation, and hydroxylamino- and amino-dinitrotoluenes became predominant metabolites (Ziganshin et al., 2010; Khilyas et al., 2013). All enzymes, which were up-regulated at the second stage of TNT transformation, retained a high level of biosynthesis at the third stage. Therefore, it could be concluded that the up-regulated enzymes identified in this study participate in the formation of all types of TNT intermediates.

A broad range of key metabolic enzymes are up-regulated during TNT transformation (**Figures 1**–**3**). It is known that toxic effects of TNT are partially attributed to reactive oxygen radicals (ROS) generated through the formation of nitro anion radicals (Spain, 1995; Kumagai et al., 2004). Furthermore, the interaction of NO and superoxide anion $O_{2}^{•-}$ generates peroxynitrite with a more disruptive power (Kumagai et al., 2004). This reaction is catalyzed by neuronal nitric oxide synthase. Peroxinitrite is a stronger oxidant than both NO and $O_{2}^{•-}$ and it might increase the oxidative stress through its binding with biomolecules (Beckman et al., 1990; Beckman and Koppenol, 1996; Vasquez-Vivar et al., 1997).

Reactive oxygen species and reactive nitrogen species (RNS) could be neutralized by three cellular mechanisms: (a) by enzymes as SOD, catalase, glutathione peroxidase, and NO dioxigenase (NOD); (b) by transition metals such as Fe II, Cu II, Mn II; and (c) by antioxidant-scavengers such as ascorbic acid, cysteine, and SH groups of plasma proteins (Ignarro et al., 2002; Yao et al., 2004). In this study, we established the defense properties of enzymatic yeast during TNT transformation. We have shown that SOD and catalase were up-regulated at all stages of TNT biotransformation. Recently, it was established that TNT is an inductor of antioxidant system in human, mouse and rat hepatoma cell lines (Naumenko et al., 2016). Of these two, the upregulated level of SOD was higher (**Figure 4**). Although the main part of nitrate occurred in yeast growth medium through the abiotic nitrite oxidation at acidic pH, the up-regulation of NOD at the second stage of TNT transformation shows that this enzyme could also participate in nitrate formation from NO and $O_{2}^{•-}$. Furthermore, we observed the activation of GST, disulfide isomerase and thioredoxin, peroxiredoxine, and thioredoxin reductases which participated in TNT stress response and oxidative stress response. The primary metabolic function of GST is the conjugation of electrophilic xenobiotic compounds followed by the efflux of the GST-xenobiotic complex from the cell (Barreto et al., 2006). An increase in the activity of disulfide isomerase located in endoplasmic reticulum and the subsequent microsome formation containing GST were found to trigger the lipid-soluble xenobiotics metabolism inside the yeast cells (Barreto et al., 2006). Thus, our data supports the concept of enzymatic neutralization of TNT-induced stress.

## Conclusion

The results of the present study show that membrane-bound oxidoreductases are the pioneering proteins that can trigger the TNT transformation via TNT monohydride-Meisenheimer complexes. In particular, NADH flavin oxidoreductases/NADH oxidases related to OYEs, and some transferases can be up-regulated at all stages of TNT transformation. The upregulation of several stress response proteins (SOD, catalase, glutathione peroxidase, and GST) was also detected. Finally, the involvement of intracellular NOD in NO formation during nitrites oxidation was established. These findings support the biogenic method of NO formation in addition to the abiotic formation pathway explored in previous studies.

## Author Contributions

IK, GL, and OI planned and performed experiments, analyzed data, contributed reagents, and wrote the paper.

## Conflict of Interest Statement

The authors declare that the research was conducted in the absence of any commercial or financial relationships that could be construed as a potential conflict of interest.

## Footnotes

1 http://www.matrixscience.com
