# Supplementary material for: Proteomic Analysis of 2,4,6-Trinitrotoluene Degrading Yeast Yarrowia lipolytica
Source: Front Microbiol. 2017 Dec 22;8:2600. doi: 10.3389/fmicb.2017.02600 (PMC5744042; doi:10.3389/fmicb.2017.02600)
Supplement: Supplementary file 1 [file Data_Sheet_1.PDF]

Supporting Information for

Proteomic analysis of 2,4,6-trinitrotoluene degrading yeast *Yarrowia lipolytica*

Irina V. Khilyas[1*], Guenter Lochnit[2], Olga N. Ilinskaya[1]

[1] Institute of fundamental medicine and biology, Kazan (Volga Region) Federal University, Kazan, Russian Federation
[2] Protein Analytics, Institute of Biochemistry, Faculty of Medicine, Justus Liebig University, Giessen, Germany

*e-mail: irina.khilyas@gmail.com

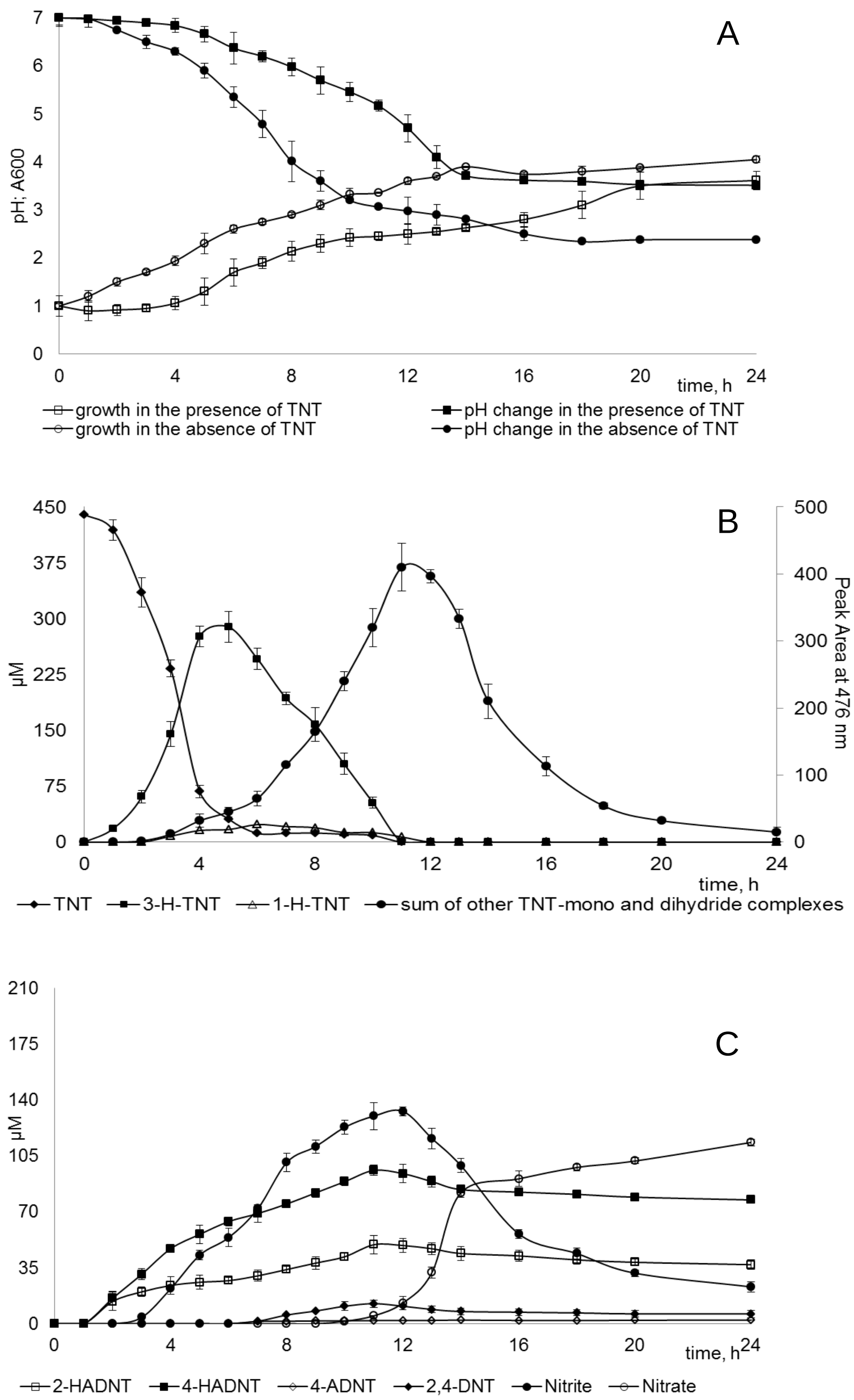

Fig. S1. (A) Growth (A_{600}) of *Y. lipolytica* VKPM Y-3492 and pH change in the presence and absence of TNT (initial medium pH 7.0). (B, C) Formation of metabolites during TNT transformation by *Y. lipolytica*. Concentrations of TNT, 3-H^--TNT, HADNTs, 4-ADNT, 2,4-DNT, NO_2^-, and NO_3^- are expressed in µM. 1-H^--TNT (peak area at 476 nm) and sum of mono- and dihydride complexes related to 3-H^--TNT are expressed as sum of absorbance peak area at 476 nm. Error bars represent one standard deviation of triplicate experiments.

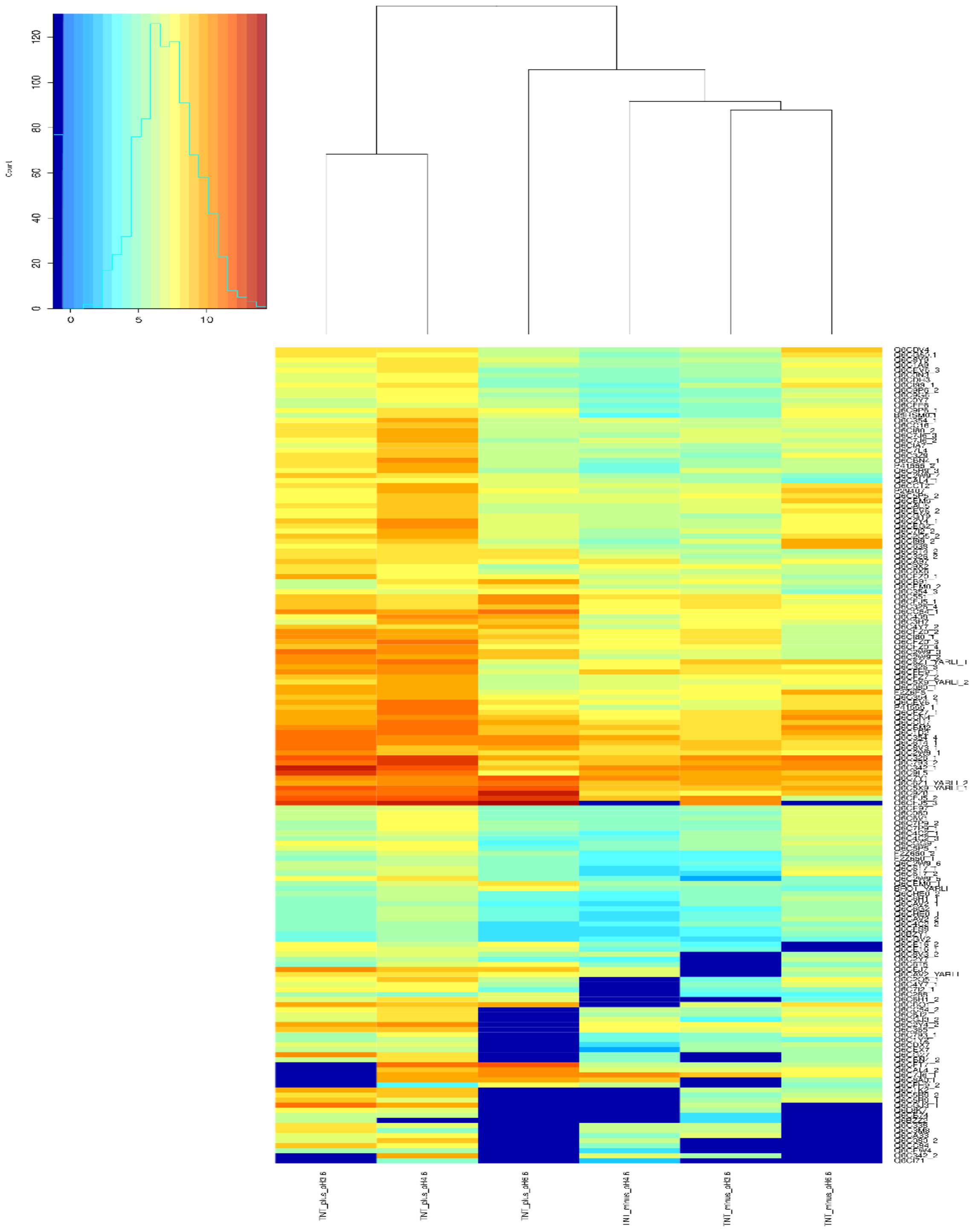

Fig. S2. List of proteins of *Y. lipolytica* are up-regulated during TNT biotransformation

Table S1. Proteome analysis of up-regulated proteins at the first stage of TNT transformation by *Y.lipolytica*.

#	PD Quest Spot no.	Uniprot Accesion Number	Identified protein	Score	Mass (kDa)	Matches	Coverage, %	Process	Localization	Family/Superfamily
1.	2511	Q6CFT7	ATP synthase subunit beta	253	54616	26	57	Metabolic Electron/proton transport, ATP synthesis	Mitochondrion, plasma membrane	ATPase alpha/beta chains family.
2.	3805	Q6C6T5	Alpha aminoadipate reductase [Komagataella pastoris GS115]	186	154766	29	30	Metabolic RedOx Biosynthetic	Cytosol	ATP-dependent AMP-binding enzyme family
3.	3813	Q6C288	26S proteasome regulatory subunit [Wickerhamomyces ciferrii]	65	102811	11	17	Proteasome Catabolic Fatty acid metabolic process	Proteasome	Proteasome/cyclosome repeat
4.	4308	Q6CC54	Disulfide isomerase [Sphaerulina musiva SO2202] / Thioredoxin [Macrophomina phaseolina MS6]	198	41132	16	47	Metabolic RedOx	Endoplasmic reticulum/ Cytosol, mitochondria	Disulfide isomerase family / Thioredoxin superfamily
5.	4310	Q6C9Z8	Piso0_000015 [Millerozyma farinosa CBS 7064]	130	37908	17	52	Metabolic	Cytosol	Rossmann superfamily
6.	4408	Q6C9L5	Pyruvate decarboxylase [Cyberlindnera jadinii]	251	63090	35	49	Metabolic	Mitochondrion	TPP enzyme family
7.	4408	Q6CFJ5	NADH flavin oxidoreductase/NADH oxidase [Fomitopsis pinicola FP-58527 SS1] Isoform 3	248	49133	33	64	RedOx	Cell membrane	Old yellow enzyme (OYE)-like FMN binding domain.
8.	4502	Q6C7Y1	ATP-citrate lyase [Aspergillus oryzae 3.042]	159	54190	22	53	Metabolic	Cytosol	ATP-grasp domain
9.	4509	Q6C354	2-methylcitrate dehydratase [Wickerhamomyces ciferrii]	144	58099	20	51	Propanoate metabolism	Cytosol, mitochondria	MmgE/PrpD family
10.	5301	Q6C365	YALI0F02255p	303	35760	23	68	Unknown	Unknown	Unknown
11.	5401	Q6CFJ5	NADH flavin oxidoreductase/NADH oxidase [Fomitopsis pinicola FP-58527 SS1] Isoform 1	338	42925	35	74	RedOx	Cell membrane	Old yellow enzyme (OYE)-like FMN binding domain.
12.	5415	Q6CFJ5	NADH flavin oxidoreductase/NADH oxidase [Fomitopsis pinicola FP-58527 SS1] Isoform 2	346	42925	35	69	RedOx	Cell membrane	Old yellow enzyme (OYE)-like FMN binding domain
13.	5725	Q6C6T4	Transketolase [Ophiostoma piceae UAMH 11346]	122	76469	13	27	Metabolic	Cytosolic	Thiamine pyrophosphate (TPP) enzyme family Transketolase family

14.	6108	Q6CB91	Glutathione S-transferase, variant [Exophiala dermatitidis NIH/UT8656]	82	25191	7	34	Glutathione metabolism Metabolic RedOx	Cytosol, Mitochondria, microsomal	GST superfamily Thioredoxin superfamily
15.	6703	Q6CEM0	Similar to Methylcrotonoyl-CoA carboxylase subunit alpha, mitochondrial	104	79740	22	37	Metabolic	Mitochondria	Carbamoyl-phosphate synthase L chain, N-terminal domain
16.	6707	Q6CEM0	Similar to Methylcrotonoyl-CoA carboxylase subunit alpha, mitochondrial	131	79740	20	29	Metabolic	Mitochondria	Carbamoyl-phosphate synthase L chain, N-terminal domain
17.	7514	Q6C4Y7	Glucose-6-phosphate 1-dehydrogenase	297	57038	39	71	Metabolic RedOx	Cytosol	Glucose-6-phosphate dehydrogenase family
18.	8111	Q6C8A0	RNA-binding domain-containing protein [Gloeophyllum trabeum ATCC 11539]	56	24702	5	34	Splicing (RNA)	Cytosol	RNA recognition motif (RRM) superfamily
19.	8217	Q6CFH9	ATP synthase subunit d, mitochondrial [Wickerhamomyces ciferrii]					Metabolic Electron/proton transport, ATP synthesis	Mitochondria	ATP synthase D chain, mitochondrial (ATP5H) family
20.	8309	Q6CAL4	Adenylate kinase	332	36638	25	72	RedOx	Mitochondria	Medium chain reductase/dehydrogenase (MDR)/zinc-dependent alcohol dehydrogenase-like family

Table S2. Proteome analysis of up-regulated proteins at the second stage of TNT transformation by *Y.lipolytica*.

#	PD Quest Spot no.	Uniprot Accesion Number	Identified protein	Score	Mass (kDa)	Matches	Coverage, %	Process	Localization	Family/Superfamily
1.	4809	Q6CFZ0	Similar to glycoside hydrolase family 63 protein [Botryotinia fuckeliana T4]	439	118925	49	48	Metabolic	Cell wall	Glycosyl hydrolase family 63
2.	4811	Q6CFZ0	Similar to glycoside hydrolase family 63 protein [Botryotinia fuckeliana T4]	255	118925	42	45	Metabolic	Cell wall	Glycosyl hydrolase family 63
3.	5801	Q6C6H1	Glutamate/phenylalanine/leucine/valine dehydrogenase [Macrophomina phaseolina MS6]	551	112538	61	58	Metabolic RedOx Catabolic	Mitochondrion	Glutamate dehydrogenases
4.	6627	Q6C3H7	Catalase	172	59970	18	36	RedOx Catabolic Stress response	Cytosol, peroxisome	Catalase family
5.	7301	Q6C438	Coproporphyrinogen III oxidase [Botryotinia fuckeliana B05.10]	123	52126	19	33	Metabolic Biosynthetic RedOx	Mitochondrion	Coproporphyrinogen oxidase family
6.	1304	Q6C080	Saccharopepsin [Wickerhamomyces ciferrii]	86	42804	10	38	Protein biogenesis (Proteolysis)	Vacuole	Family/Superfamily
7.	2314	Q6C8Y9	Acyl-CoA dehydrogenase-like protein [Chaetomium thermophilum var. thermophilum DSM 1495]	295	46148	26	53	Metabolic, RedOx,	Mitochondrion	peptidase A1 family
8.	2403	Q6CDV4	ATP-dependent RNA helicase	213	44602	20	46	RNA biogenesis (translation), Catabolic	Cytosol	acyl-CoA dehydrogenase family
9.	2613	Q6C342	Heat shock protein 60, mitochondrial precursor (HSP 60) [Meyerozyma guilliermondii ATCC 6260]	376	60799	40	67	Metabolic, Protein biogenesis (protein folding)	Cytosol	DEAD box helicase family. eIF4A subfamily
10.	2702	Q6CCN4	ATP-dependent molecular chaperone HSC82 [Candida tropicalis MYA-3404]	432	80285	49	55	Stress response, Protein biogenesis (protein folding)	Cytosol. Mitochondrion	chaperonin (HSP60) family
11.	2712	Q6C1Y2	Heat shock protein 78, mitochondrial precursor HSP78 [Candida tenuis ATCC 10573]	231	87588	30	46	Metabolic, Stress response, Catabolic	Mitochondrion	heat shock protein 90 family
12.	3303	Q6CI71	Piso0_004301 [Millerozyma farinosa CBS 7064]	141	41945	14	45			clpA/clpB family
13.	3409	Q6CGY9	Similar to Mitochondrial-processing peptidase subunit beta; acc. no.	249	52754	21	47	Metabolic, proteolysis	Mitochondrion	

			P11913 [Pyronema omphalodes CBS 100304]							
14.	3410	Q6C7P9	Argininosuccinate lyase [Wickerhamomyces ciferrii]	257	52078	56	52	Biosynthetic	Cytosol	Peptidase M16 family
15.	3510	Q6C1A9	Glutamate dehydrogenase	290	50273	30	71	Metabolic, RedOx	Mitochondrion	lyase 1 family
16.	3601	Q6CFF6	Alpha-isopropylmalate synthase/homocitrate synthase (LEU4) [Scheffersomyces stipitis CBS 6054]	424	64576	38	66	Metabolic, biosynthetic	Mitochondrion	Glu/Leu/Phe/Val dehydrogenases family
17.	4315	Q6CEX7	Methylenetetrahydrofolate dehydrogenase [NAD+] [Wickerhamomyces ciferrii]	173	36470	14	46	RedOx, biosynthetic	Mitochondrion	alpha-IPM synthase/homocitrate synthase family. LeuA type 2 subfamily.
18.	4408	Q6CEM5	S-adenosylmethionine synthase	279	42383	28	72	Metabolic, biosynthetic	Cytosol	tetrahydrofolate dehydrogenase/cyclohydrolase family
19.	4414	Q6CAT7	Elongation factor 1 gamma domain-containing protein (EF 1)	328	58099	13	70	RNA biogenesis (Translation elongation factor activity)	Cytosol	AdoMet synthase family
20.	4520	Q6C354	2-methylcitrate dehydratase [Wickerhamomyces ciferrii]	378	99528	34	45	Propanoate metabolism	Cytosol mitochondria	Glutathione S-transferase C-terminal-like, alpha helical domain of the Gamma subunit of Elongation Factor 1B and similar proteins
21.	4708	Q6C4C2	Piso0_004415 [Millerozyma farinosa CBS 7064]	305	112538	48	46	RNA biogenesis (Metabolic, regulation of transcription, DNA-templated)	Cytosol	MmgE/PrpD family
22.	4804	Q6C6H1	Glutamate/phenylalanine/leucine/valine dehydrogenase [Macrophomina phaseolina MS6]	234	131113	5	34	Metabolic, RedOx, catabolic	Mitochondrion	clpA/clpB family
23.	4813	Q6CAV2	Pyruvate carboxylase	541	131113	33	57	Metabolic, gluconeogenesis	Mitochondrion	Glu/Leu/Phe/Val dehydrogenases family
24.	4816	Q6CAV2	Pyruvate carboxylase	82	109224	59	15	Metabolic, gluconeogenesis	Mitochondrion	
25.	4820	Q6CHE0	Similar to Putative glycine dehydrogenase [decarboxylating], mitochondrial; acc. no. Q09785 [Pyronema omphalodes CBS 100304]	152	43816	11	43	Metabolic, RedOx, catabolic	Mitochondrion	
26.	5413	Q6C0Y7	pyruvate dehydrogenase E1 component [Colletotrichum higginsianum]	177	58158	19	52	Metabolic, RedOx, glycolysis	Mitochondrion	GcvP family
27.	5506	Q6C6J2	cystathionine beta-synthase	190	70322	22	42	Biosynthetic	Cytosol	PDK family kinases

			[Candida albicans WO-1]							
28.	5514	Q6C2W9	retinal dehydrogenase 2 [Byssochlamys spectabilis No. 5]	325	85266	31	37	Metabolic, RedOx,	Microsomal or cytosolic	
29.	5603	Q6C9G6	Similar to Probable succinate dehydrogenase [ubiquinone] flavoprotein subunit, mitochondrial; acc. no. Q9UTJ7 [Pyronema omphalodes CBS 100304]	494	85909	51	69	TCA, RedOx, electron/proton transport	mitochondrial	aldehyde dehydrogenase family
30.	5708	Q6C9P6	Mitochondrial aconitate hydratase [Yarrowia lipolytica]	395	100644	42	51	TCA, metabolic	mitochondrial	FAD-dependent oxidoreductase 2 family. FRD/SDH subfamily.
31.	5710	Q6C638	5-methyltetrahydropteroyltriglutamate--homocysteine methyltransferase [Ogataea parapolymorpha DL-1]	223	85266	28	31	Biosynthetic	mitochondrial	Aconitase family
32.	5713	F2Z650	formate-tetrahydrofolate ligase [Exophiala dermatitidis NIH/UT8656]	213	109224	31	35	RedOx, Biosynthetic	mitochondrial	vitamin-B12 independent methionine synthase family.
33.	5715	Q6C9P6	Mitochondrial aconitate hydratase	174	100644	19	25	TCA, metabolic	mitochondrial	formate--tetrahydrofolate ligase family
34.	5801	Q6CHE0	Similar to Putative glycine dehydrogenase [decarboxylating], mitochondrial; acc. no. Q09785 [Pyronema omphalodes CBS 100304]	230	117762	29	27	Metabolic, RedOx, catabolic	Mitochondrion	Aconitase family
35.	5809	F2Z650	formate-tetrahydrofolate ligase [Exophiala dermatitidis NIH/UT8656]	257	117762	44	40	RedOx, Biosynthetic	mitochondrial	GcvP family
36.	5811	Q6C617	elongation factor 3 [Meyerozyma guilliermondii ATCC 6260] (EF 3)	131	36774	13	43	Metabolic, catabolic	Cytosol	formate--tetrahydrofolate ligase family
37.	5812	Q6C617	elongation factor 3 [Meyerozyma guilliermondii ATCC 6260] (EF 3)	209	46393	27		Metabolic, catabolic	Cytosol	ABC transporter superfamily Uup; ATPase components of ABC transporters with duplicated ATPase domains
38.	6313	Q6CEW4	NADPH quinone reductase [Aspergillus oryzae 3.042]	123	37772	14	49	RedOx	mitochondrion	ABC transporter superfamily Uup; ATPase components of ABC transporters with duplicated ATPase domains
39.	6403	Q6C2Y4	Isocitrate dehydrogenase [NADP]	136	40092	15	63	TCA, metabolic, RedOx	mitochondrion	zinc-containing alcohol dehydrogenase family. Quinone oxidoreductase

										subfamily
40.	7301	Q6CDX7	putative cell wall biogenesis protein glutathione transferase protein [Neofusicoccum parvum UCRNP2]	206	3591 1	25	63	Glutathione metabolism ,	cell wall	isocitrate and isopropylmalate dehydrogenases family
41.	7302	Q6CA33	YALI0D06303p	206	5173 4	20		TCA, RedOx	Unknown	
42.	7315	Q6CCU7	Glyceraldehyde-3-phosphate dehydrogenase	40	1497 8	4	25	Metabolic, RedOx, glycolysis	mitochondr ion	isocitrate and isopropylmalate dehydrogenases family.
43.	7409	Q6C793	Citrate synthase	181	5576 7	19	30	TCA, Metabolic	mitochondr ion	glyceraldehyde-3-phosphate dehydrogenase family.
44.	7604	B5RSM0	YALI0F21494p	162	3131 8	18	54	Unknown	Unknown	citrate synthase family
45.	1511	Q6CGJ3	carboxypeptidase C [Wickerhamomyc es ciferrii]	437	7006 2	43	62	Protein biogenesis (Proteolysis)	vacuole	
46.	2303	F2Z6F8	14-3-3 protein [Sphaerulina musiva SO2202]	297	6631 0	22	36	Signal transductio n, apoptotic cell death, cell cycle control	Cytosol	Serine carboxypeptidase
47.	2606	Q6CBM2	Chaperone protein [Wickerhamomyc es ciferrii]	402	1133 23	40	48	Protein biogenesis (Protein folding)	Endoplasmi c reticulum	Tetratrico Peptide Repeat (TPR) superfamily
48.	2612	Q6CIA7	cytoplasmic ATPase that is a ribosome-associated molecular chaperone, putative; heat shock protein, putative [Candida dubliniensis CD36]	160	7976 1	21	37	Proton transport	Cytosol	heat shock protein 70 family
49.	2802	Q6C6V1	Ubiquitin-activating enzyme E1 1 [Ogataea parapolymorpha DL-1]	190	7032 2	22	42	Protein biogenesis (protein modificatio n)	Proteasome	heat shock protein 70 family
50.	2805	Q6CGG9	Coronin	325	8526 6	31	37	cytoskeleto n remodeling, and regulation of vesicle trafficking	Cytosol, Cell surface,	ubiquitin-activating E1 family
51.	3301	Q6C7L4	Thioredoxin reductase	204	3445 0	12	61	RedOx, stress response	Cytosol	WD repeat coronin family
52.	3414	Q6CEV6	elongation factor 1 gamma domain-containing protein (EF1)	152	4508 9	11	27	RNA biogenesis (translation elongation factor activity)	Cytosol	class-II pyridine nucleotide-disulfide oxidoreductase family
53.	3627	Q6CDH3	Polyadenylate-binding protein, cytoplasmic and nuclear	357	6940 1	43	64	RNA biogenesis (regulation of translation)	Cytosol Nucleus	Glutathione S-transferase C-terminal-like, alpha helical domain of the Gamma subunit of Elongation Factor 1B and similar proteins
54.	3803	Q6CFS9	alanyl-tRNA synthetase [Geotrichum	133	1050 26	27	33	RNA biogenesis (regulation	Cytosol	polyadenylate-binding protein type-1 family

			candidum]					of translation)		
55.	3809	Q6CGV2	Histidine biosynthesis trifunctional protein [Ogataea parapolymorpha DL-1]	207	92340	21	35	Metabolic, RedOx, biosynthetic	Endoplasmatic reticulum	Class II tRNA amino-acyl synthetase-like catalytic core domain
56.	3821	Q6BZY1	phosphoribosylformylglycinamidine synthase [Wickerhamomyces ciferrii]	230	142517	32	29	Biosynthetic	cytoplasma	histidinol dehydrogenase family
57.	4308	Q6C9Z8	YALI0D07062p	146	37908	16	45	Metabolic	Unknown	FGAMS family
58.	4313	Q6CEV6	elongation factor 1 gamma domain-containing protein	236	41420	21	49	RNA biogenesis (translation elongation factor activity)	Cytoplasm	Rossmann-fold NAD(P)(+)-binding proteins aldehyde reductase
59.	4405	Q6CEV6	elongation factor 1 gamma domain-containing protein	203	45089	17	45	RNA biogenesis (translation elongation factor activity)	Cytoplasm	Glutathione S-transferase C-terminal-like, alpha helical domain of the Gamma subunit of Elongation Factor 1B and similar proteins
60.	4511	Q6C7J6	acetaldehyde dehydrogenase [Issatchenkia terricola]	300	56830	32	66	Metabolic, RedOx	Microsomes, mitochondria and peroxisomes	Glutathione S-transferase C-terminal-like, alpha helical domain of the Gamma subunit of Elongation Factor 1B and similar proteins
61.	4513	Q6C354	2-methylcitrate dehydratase [Wickerhamomyces ciferrii]	295	58099	28	57	Propanoate metabolism	Cytosol mitochondria	aldehyde dehydrogenase family
62.	4523	Q6C7J6	acetaldehyde dehydrogenase [Issatchenkia terricola]	402	56830	29	64	Metabolic, RedOx	Microsomes, mitochondria and peroxisomes	MmgE/PrpD family
63.	4608	Q6CBN4	phosphoenolpyruvate carboxykinase-like protein [Chaetomium thermophilum var. thermophilum DSM 1495]	373	62513	37	64	gluconeogenesis	Cytosolic and mitochondrial.	aldehyde dehydrogenase family
64.	4703	Q6C4C2	Piso0_004415 [Millerozyma farinosa CBS 7064]	141	99528	18	22	RNA biogenesis (Metabolic, regulation of transcription, DNA-templated)	Cytosol	Phosphoenolpyruvate carboxykinase (PEPCK)
65.	4705	Q6C4C2	Piso0_004415 [Millerozyma farinosa CBS 7064]	503	99528	57	58	RNA biogenesis (Metabolic, regulation of transcription, DNA-templated)	Cytosol	clpA/clpB family
66.	4824	Q6CB74	Yhb1 nitric oxide dioxygenase [Candida orthopsilosis Co 90-125]	211	41420	17	38	RedOx, oxygen transport	Cytoplasma	clpA/clpB family
67.	5310	Q6C365	YALI0F02255p	303	3576	23	68	Unknown	Unknown	globin family

					0					
68.	5507	Q6C3Z9	Acetyl-CoA hydrolase	398	6018 2	35	66	Metabolic	Cytoplasma	
69.	5517	P41555	Isocitrate lyase	208	5830 0	20	36	TCA, metaboli glyoxylate cyclec,	Glyoxysom e, Peroxisome	acetyl-CoA hydrolase/transferas e family
70.	5807	Q6C6G2	YALI0E09801p	51	3605 4	10	31	Metabolic, RedOx	Unknown	isocitrate lyase/PEP mutase superfamily. Isocitrate lyase family.
71.	6303	Q6CAU5	Pyridoxine biosynthesis protein PDX1 [Mucor circinelloides f. circinelloides 1006PhL]	244	3233 9	23	52	Metabolic, biosyntheti c	Cytosol	NAD dependent epimerase/dehydrata se family
72.	6309	Q6CI80	potassium channel subunit (predicted) [Schizosaccharom yces pombe 972h-]	340	3935 4	29	73	Ionic transfer	Cell membrane	PdxS/SNZ family.
73.	6315		isocitrate dehydrogenase subunit 1 [Lipomyces starkeyi]	181	3998 1	17	41	TCA	Mitochondr ion	Aldo/keto reductase family
74.	6402	Q6C2Y4	Isocitrate dehydrogenase [NADP]	237	4639 3	31	56	TCA, metabolic, RedOx	Mitochondr ion	isocitrate and isopropylmalate dehydrogenases family.
75.	6410	Q6C793	Citrate synthase	265	5173 4	25	66	TCA, Metabolic	Mitochondr ion	isocitrate and isopropylmalate dehydrogenases family
76.	6412	Q6C7I2	Citrate synthase	296	5111 6	31	54	TCA, Metabolic	Mitochondr ion	citrate synthase family
77.	6417	Q6C7I2	Citrate synthase	133	5111 6	20	41	TCA, Metabolic	Mitochondr ion	citrate synthase family
78.	6501	Q6C2W9	retinal dehydrogenase 2 [Byssochlamys spectabilis No. 5]	335	5649 7	43	70	Metabolic, RedOx,	Microsomal or cytosolic	citrate synthase family
79.	6504	Q6CE97	Adenylosuccinate lyase [Ogataea parapolymorpha DL-1]	305	5457 2	27	54	Biosyntheti c	Cytosol	aldehyde dehydrogenase family
80.	6505	P41555	Isocitrate lyase	431	6018 2	37	66	TCA, metaboli glyoxylate cyclec,	Glyoxysom e, Peroxisome	lyase 1 family. Adenylosuccinate lyase subfamily.
81.	6604	Q6C2Q5	acetate--CoA ligase	311	7255 2	40	57	Metabolic, biosyntetic	Mitochondr ion	isocitrate lyase/PEP mutase superfamily. Isocitrate lyase family.
82.	6605	Q6C2Q5	acetate--CoA ligase	419	7255 2	22	35	Metabolic, biosyntetic	Mitochondr ion	Ligases family
83.	6711	Q6CI99	classical protein kinase C [Komagataella pastoris CBS 7435]	249	9402 7	27	33	Catabolic	Cytosol	Ligases family
84.	6716	Q6CI99	classical protein kinase C [Komagataella pastoris CBS 7435]	385	9402 7	39	48	Catabolic	Cytosol	PKC family
85.	6807	Q6C3M8	2-oxoglutarate dehydrogenase, mitochondrial [Ogataea parapolymorpha DL-1]	323	1141 11	49	50	TCA, metabolic, RedOx	Mitochondr ion	PKC family
86.	6811	Q6C3M8	2-oxoglutarate dehydrogenase,	429	1141 11	29	35	TCA, metabolic,	Mitochondr ion	alpha-ketoglutarate dehydrogenase

			mitochondrial [Ogataea parapolymorpha DL-1]					RedOx		family.
87.	7312	Q6CFZ7	Aspartate aminotransferase	260	4778 7	27	60	metabolic, biosyntheti c	Cytosol, mitochondr ial and chloroplasti c isozymes	alpha-ketoglutarate dehydrogenase family.
88.	7316	Q6CC16	glycerol dehydrogenase	210	3495 9	14	46	RedOx	Cytosol,	class-I pyridoxal-phosphate-dependent aminotransferase family
89.	7318	Q6CFZ7	Aspartate aminotransferase	334	4778 7	28	54	metabolic, biosyntheti c	Cytosol, mitochondr ial and chloroplasti c isozymes	glycerol-1-phosphate dehydrogenase family
90.	7404	Q6CCT2	Fumarate hydratase, mitochondrial [Ogataea parapolymorpha DL-1]	283	5311 9	26	40	TCA, metabolic	Mitochondr ial	class-I pyridoxal-phosphate-dependent aminotransferase family
91.	7408	Q6C5P5	Serine hydroxymethyltra nsferase	288	5189 3	35	56	metabolic	Cytosol, mitochondr ial	class-II fumarase/aspartase family. Fumarase subfamily.
92.	7411	Q6C5P5	Serine hydroxymethyltra nsferase	381	5189 3	36	59	metabolic	Cytosol, mitochondr ial	SHMT family
93.	7412	P29407	Phosphoglycerate kinase	356	4491 6	33	76	Glycolysis, Phosphorila tion	Cytosol	SHMT family
94.	7502	Q6C326	ATP synthase subunit alpha	388	5810 0	29	58	ATP synthesis Hydrogen ion transport Ion transport	Mitochondr ial	phosphoglycerate kinase family.
95.	7503	Q6C4Y7	Glucose-6-phosphate 1-dehydrogenase	276	5703 8	28	57	Metabolic RedOx	Cytosol	ATPase alpha/beta chains family
96.	7505	Q6C326	ATP synthase subunit alpha	426	5810 0	35	63	ATP synthesis Hydrogen ion transport Ion transport	Mitochondr ial, plasma membrane	Glucose-6-phosphate dehydrogenase family
97.	8117	Q6CFH9	ATP synthase subunit d, mitochondrial [Wickerhamomyc es ciferrii]	145	1980 8	10	59	ATP synthesis Hydrogen ion transport Ion transport	Mitochondr ial, plasma membrane	ATPase alpha/beta chains family
98.	8206	Q6C1D2	outer mitochondrial membrane protein porin [Talaromyces stipitatus ATCC 10500]	319	2962 7	19	74	regulation anion transport	Mitochondr ial	ATPase alpha/beta chains family
99.	8502	Q6C5R9	Malate synthase	242	6161 8	30	54	TCA, glyoxylate cycle	Glyoxysom e	Eukaryotic porin family
100.	8504	Q6C2Y7	hypothetical protein AOL_s00140g100 [Arthrobotrys oligospora ATCC	119	4678 0	11	27	Unknown	Unknown	malate synthase family.

			24927]							
101.	8505	Q6C5R9	Malate synthase	429	6161 8	46	56	TCA, glyoxylate cycle	Glyoxysom e	
102.	8507	Q6C060	Formation of crista junctions protein 1 OS=Yarrowia lipolytica (mitochondrial inner membrane)	221	5984 5	17	29	Protein biogenesis (protein biogenesis)	Mitochondr ion inner membrane; Single-pass membrane protein	malate synthase family.

Table S3. Proteome analysis of up-regulated proteins at the third stage of TNT transformation by *Y.lipolytica*.

#	PD Quest Spot no.	Uniprot Accesion Number	Identified protein	Score	Mass (kDa)	Matches	Coverage, %	Process	Localization	Family/Superfamily
1.	1405	Q6C080	Saccharopepsin [Wickerhamomyces ciferrii]	116	42804	10	31	proteolysis	Vacuole	peptidase A1 family
2.	1611	Q6CG27	Carboxypeptidase Y [Candida maltosa Xu316]	115	62533	15	20	proteolysis	Vacuole	Serine carboxypeptidase
3.	2103	Q6BZZ2	Sulfurtransferase (Rhodanese)	79	21658	7	38	Metabolic	Cytosolic, perinuclear region	Transferases family
4.	2112	Q6CEJ7	Peroxiredoxin TSA1 [Clavispora lusitaniae ATCC 42720]	231	21543	14	61	RedOx	Cytosolic	AhpC/TSA family.
5.	2327	Q6CC94	Sphingolipid long chain base-responsive protein [Wickerhamomyces ciferrii]	178	34617	14	63		Lipid droplet, eisosome	
6.	2409	Q6CA97	Succinyl-CoA synthetase beta subunit [Macrophomina phaseolina MS6]	166	47280	25	60	Metabolic	MITO	Succinate/malate CoA ligase beta subunit family.
7.	2503	Q6CGJ3	Carboxypeptidase C [Wickerhamomyces ciferrii]	165	55767	20	24	Proteolysis	Vacuole	Serine carboxypeptidase
8.	2615	Q6C9L5	Pyruvate decarboxylase [Cyberlindnera jadinii]	316	63090	33	48	Metabolic	MITO	TPP enzyme family
9.	3324	Q6C1K2	Transaldolase	217	35868	21	45	Metabolic, pentose-phosphate shunt	Cytosolic	Transferases
10.	3601	Q6C342	Heat shock protein 60, mitochondrial precursor [Meyerozyma guilliermondii ATCC 6260]	151	60799	20	43	Metabolic, protein folding	Cytosolic c	chaperonin (HSP60) family
11.	4802	Q6CFZ0	Similar to glycoside hydrolase family 63 protein [Botryotinia fuckeliana T4]	331	118925	30	34	Metabolic, cell wall biogenesis, vesicle-mediated transport	Cell membrane	glycoside hydrolases family (GH63)
12.	4806	Q6CFZ0	Similar to glycoside hydrolase family 63 protein [Botryotinia fuckeliana T4]	498	118925	53	47	Metabolic, cell wall biogenesis, vesicle-mediated transport	Cell membrane	glycoside hydrolases family (GH63)
13.	4826	Q6CE16	Fatty acid synthase alpha subunit [Candida tropicalis MYA-3404]	94	202170	14	9	Metabolic	Cytosolic	fungal fatty acid synthetase subunit alpha family.
14.	5104	Q6CBQ1	Superoxide dismutase	81	23092	6	26	Metabolic, RedOx	Peroxisome	iron/manganese superoxide dismutase family
15.	5610	Q6C6T4	Transketolase [Ophiostoma piceae UAMH 11346]	122	76469	13	27	Metabolic	Cytosolic	Thiamine pyrophosphate (TPP) enzyme family Transketolase family

16.	6210	Q6C9X2	Malate dehydrogenase	182	35845	21	64	Metabolic, RedOx	MITO	
17.	6313	Q6C2W9	Retinal dehydrogenase 2 [Byssochlamys spectabilis No. 5]	453	56497	44	72	Metabolic, RedOx,	Microsomal or Cytosolic	aldehyde dehydrogenase family
18.	6318	Q6CI80	Potassium channel subunit (predicted) [Schizosaccharomyces pombe 972h-]	258	39354	22	67	Ionic transfer	cell membranes	Aldo/keto reductase family NAD(P)(H) oxidoreductases
19.	6501	Q6C2W9	Retinal dehydrogenase 2 [Byssochlamys spectabilis No. 5]	335	56497	43	70	Metabolic, RedOx,	Microsomal or Cytosolic	aldehyde dehydrogenase family
20.	6507	Q6C2W9	Retinal dehydrogenase 2 [Byssochlamys spectabilis No. 5]	546	56497	47	76	Metabolic, RedOx,	Microsomal or Cytosolic	aldehyde dehydrogenase family
21.	6513	Q6C2W9	Retinal dehydrogenase 2 [Byssochlamys spectabilis No. 5]	453	56497	44	72	Metabolic, RedOx,	Microsomal or Cytosolic	aldehyde dehydrogenase family
22.	6522	Q6C326	ATP synthase subunit alpha	180	58100	19		ATP synthesis Ionic transfer	MITO	ATPase alpha/beta chains family
23.	7307	Q6C8V3	Malate dehydrogenase	242	35853	16	48	TCA, metabolic, RedOx	MITO	LDH/MDH superfamily. MDH type 1 family
24.	7515	Q6C326	Dihydrolipoyl dehydrogenase	243	58100	33	60	ATP synthesis Ionic transfer	MITO	ATPase alpha/beta chains family
25.	8511	Q6C5R9	Malate synthase	393	61618	42	55	TCA, glyoxylate cycle	glyoxysome	malate synthase family.
26.	8808	Q6C3M8	2-oxoglutarate dehydrogenase, mitochondrial [Ogataea parapolymorpha DL-1]	113	114111	20	27	TCA, metabolic, RedOx	MITO	alpha-ketoglutarate dehydrogenase family.
27.	2515	Q6C0N3	Threonine synthase [Wickerhamomyces ciferrii]	120	55749	17	37	metabolic	Cytosolic	Tryptophan synthase beta superfamily (fold type II)
28.	4420	Q6CG80	tfdA family taurine dioxygenase [Aspergillus niger CBS 513.88]	126	41348	14	43	RedOx	Cytosolic	
29.	4605	Q6CBN4	phosphoenolpyruvate carboxykinase-like protein [Chaetomium thermophilum var. thermophilum DSM 1495]	388	62513	27	62	gluconeogenesis	Cytosolic and MITO	Phosphoenolpyruvate carboxykinase (PEPCK)
30.	3205	Q6C338	ATP synthase subunit gamma	144	32374	16	39	Metabolic Electron/proton transport, ATP synthesis	MITO	ATPase alpha/beta chains family.
31.	8407	Q6L8K7	Acetyl-CoA acetyltransferase	159	41560	14	48	Metabolic	peroxisome	thiolase family.
