# Supplementary material for: Proteomic Analysis of 2,4,6-Trinitrotoluene Degrading Yeast Yarrowia lipolytica
Source: Front Microbiol. 2017 Dec 22;8:2600. doi: 10.3389/fmicb.2017.02600 (PMC5744042; doi:10.3389/fmicb.2017.02600)

Supporting Information for

Proteomic analysis of 2,4,6-trinitrotoluene degrading yeast *Yarrowia lipolytica*

Irina V. Khilyas[1*], **Guenter Lochnit**[2], **Olga N. Ilinskaya**[1]

[1] Institute of fundamental medicine and biology, Kazan (Volga Region) Federal University, Kazan, Russian Federation

[2] Protein Analytics, Institute of Biochemistry, Faculty of Medicine, Justus Liebig University, Giessen, Germany

*e-mail: irina.khilyas@gmail.com

Two-dimensional gel electrophoresis (2DE) of proteins of *Yarrowia lipolytica* in the presence/absence of 2,4,6-trinitrotoluene (TNT) 1 repetition

Membranes proteins

TNT-untreated proteins of *Y.lipolytica*, pH 6.6

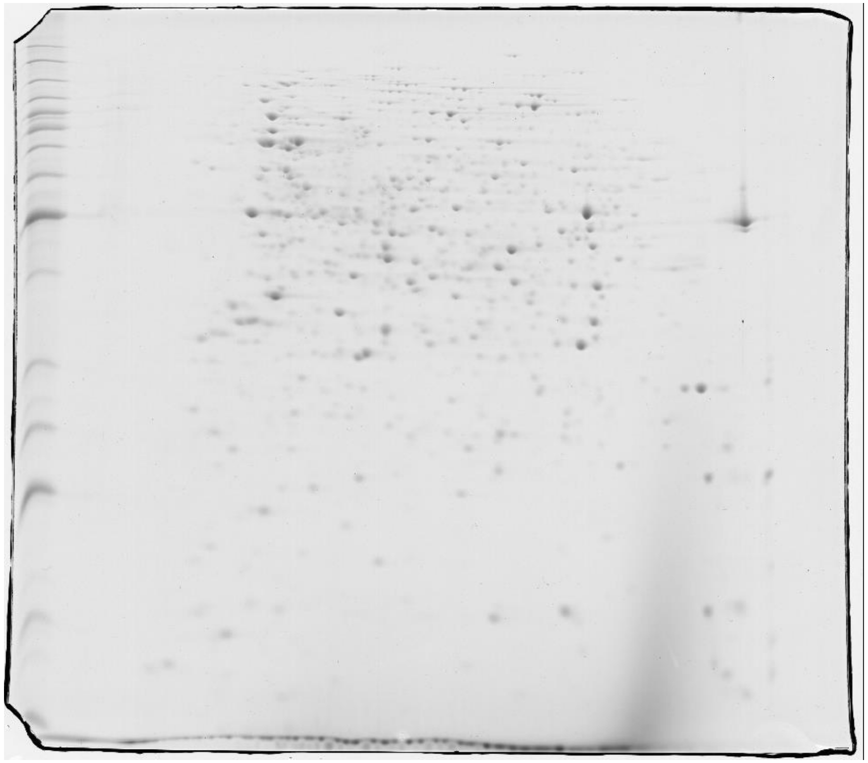

1 stage of TNT transformation, pH 6.6

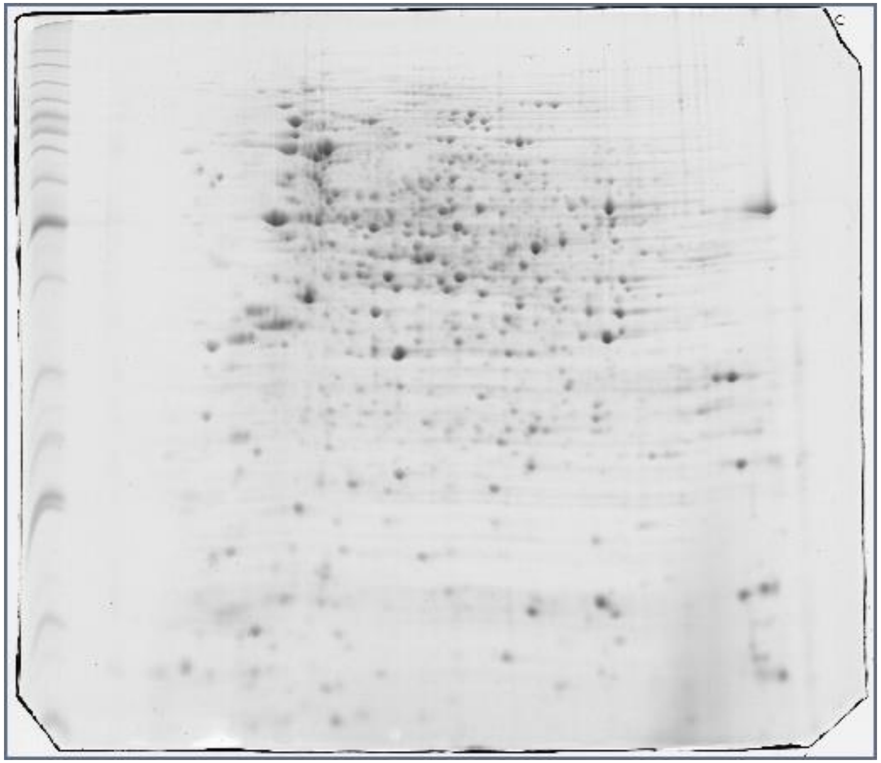

Membranes proteins

TNT-untreated proteins of *Y.lipolytica*, pH 4.6

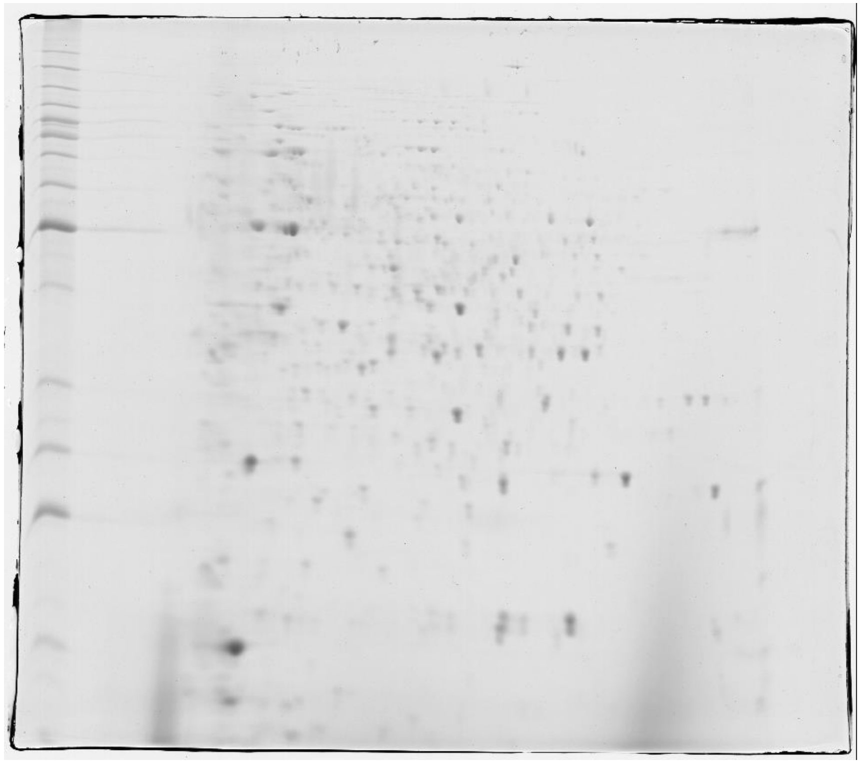

2 stage of TNT transformation, pH 4.6

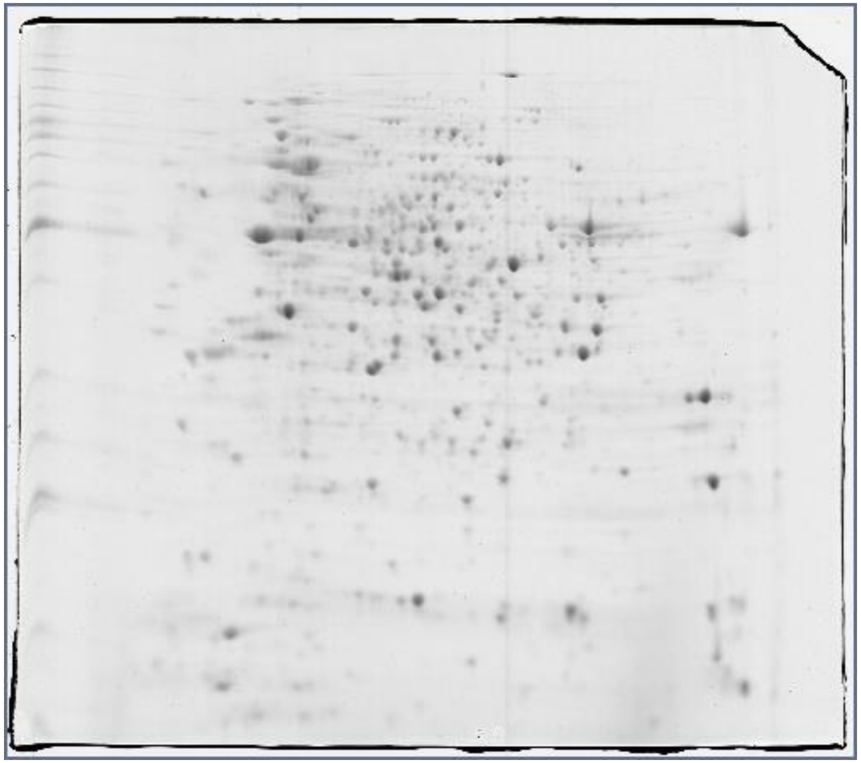

Membranes proteins

TNT-untreated proteins of *Y.lipolytica*, pH 3.6

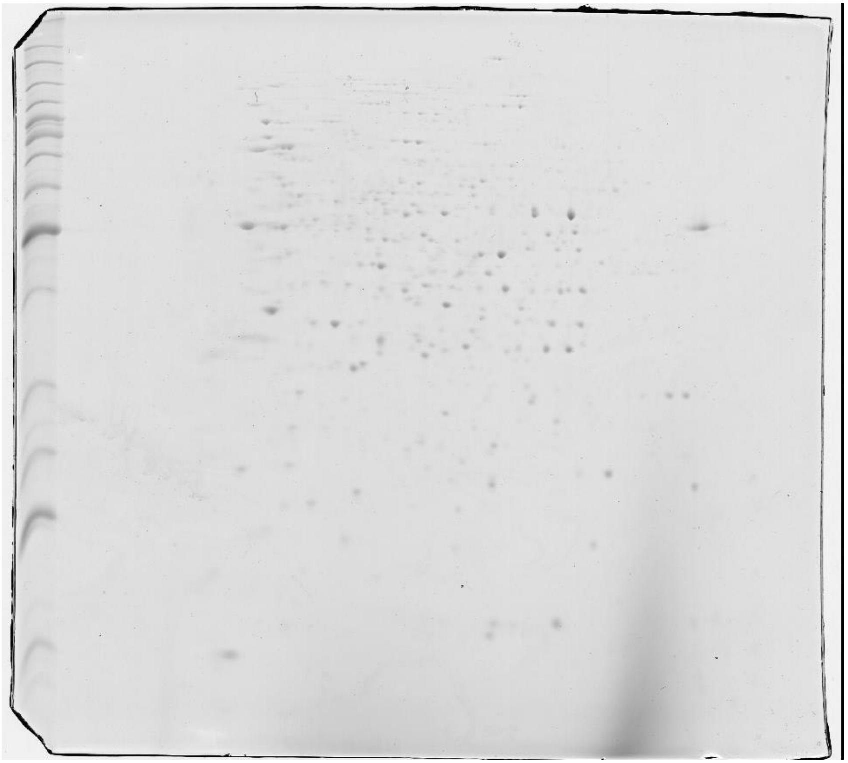

3 stage of TNT transformation, pH 3.6

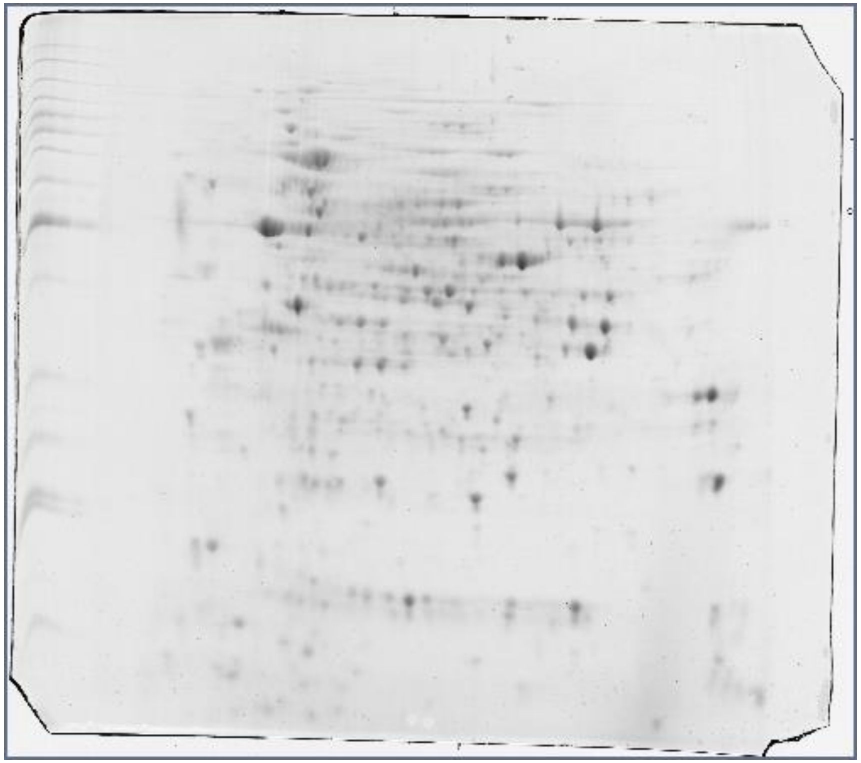

Cytosolic proteins

TNT-untreated proteins of *Y.lipolytica*, pH 6.6

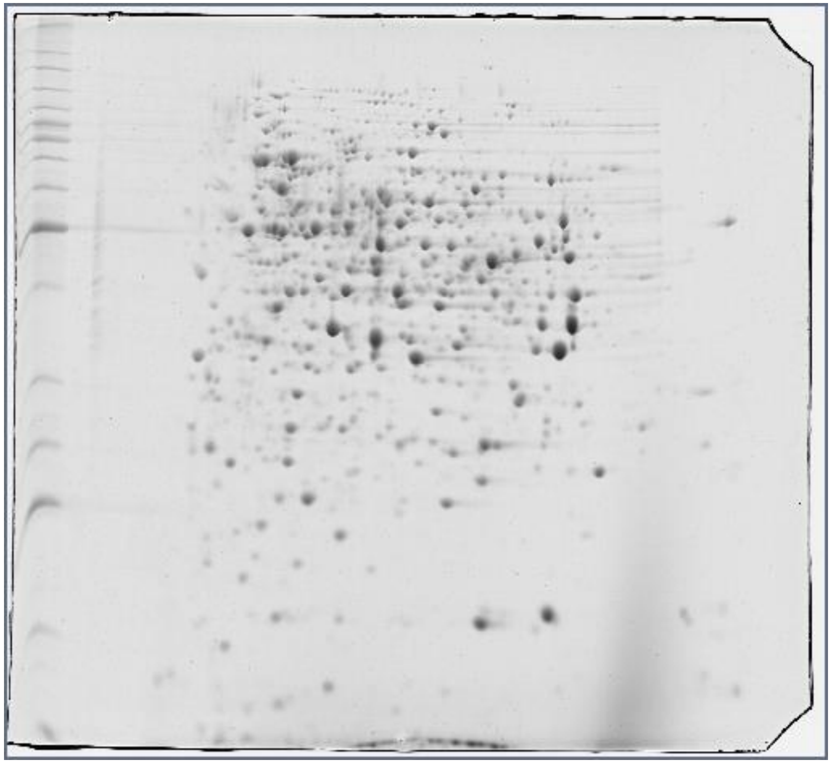

1 stage of TNT transformation, pH 6.6

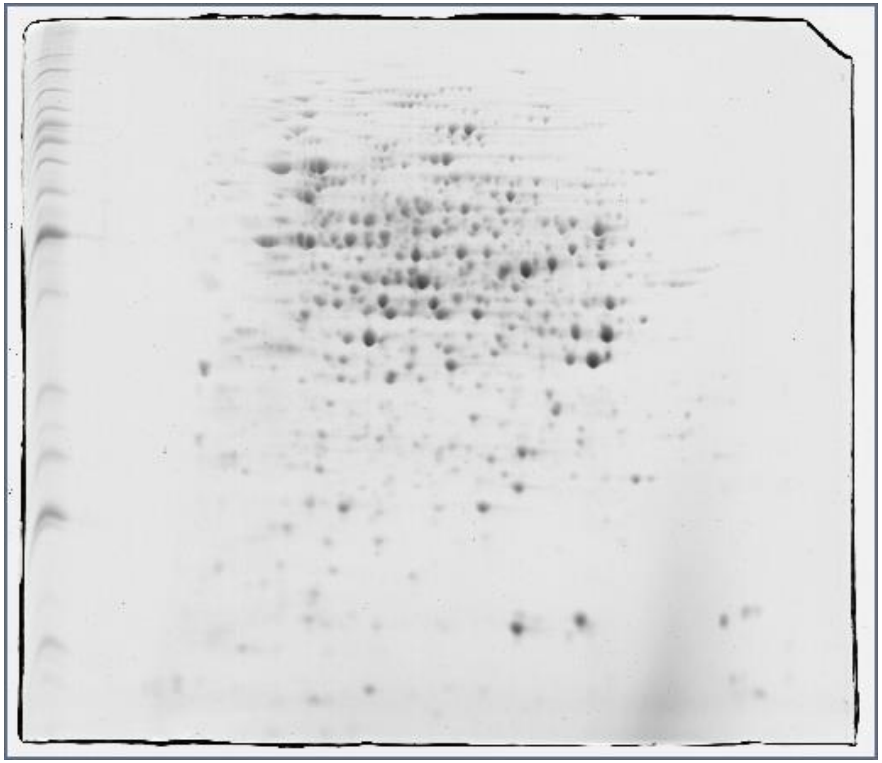

Cytosolic proteins

TNT-untreated proteins of *Y.lipolytica*, pH 4.6

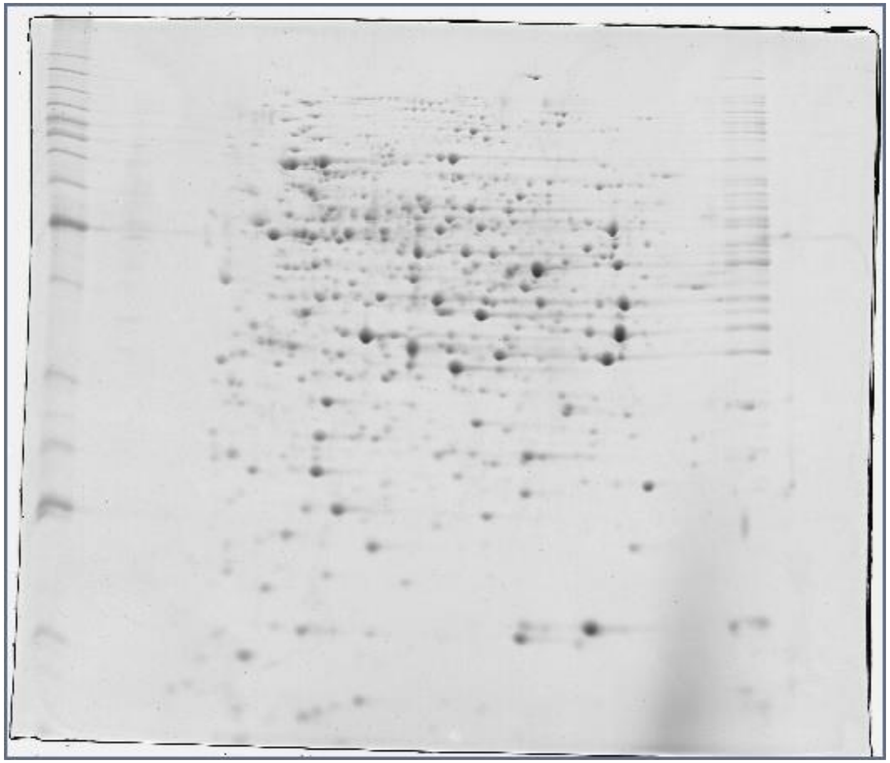

2 stage of TNT transformation, pH 4.6

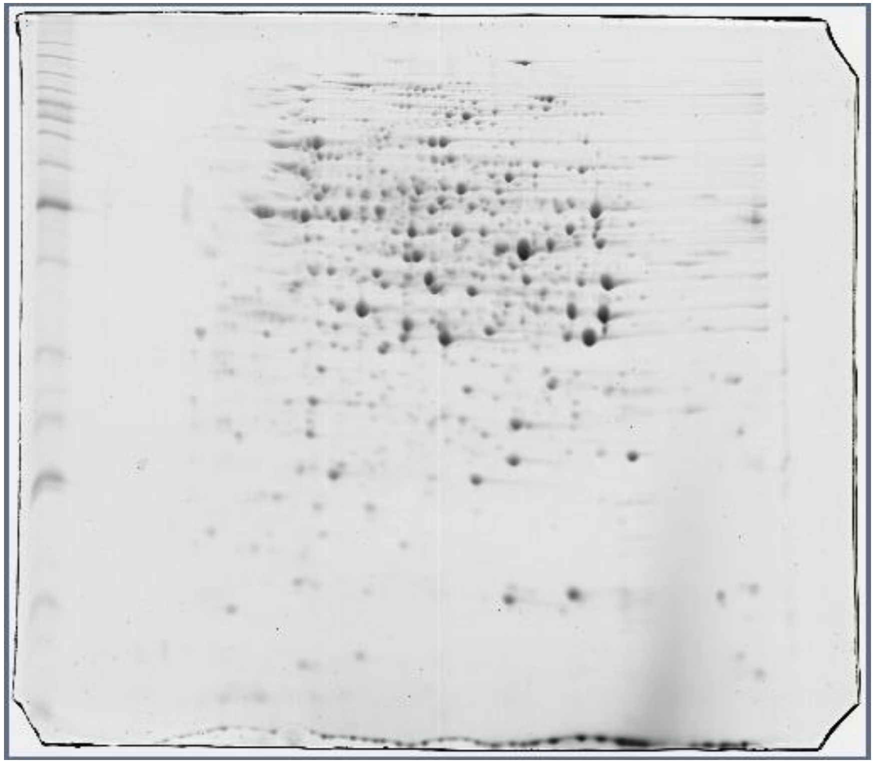

Cytosolic proteins

TNT-untreated proteins of *Y.lipolytica*, pH 3.6

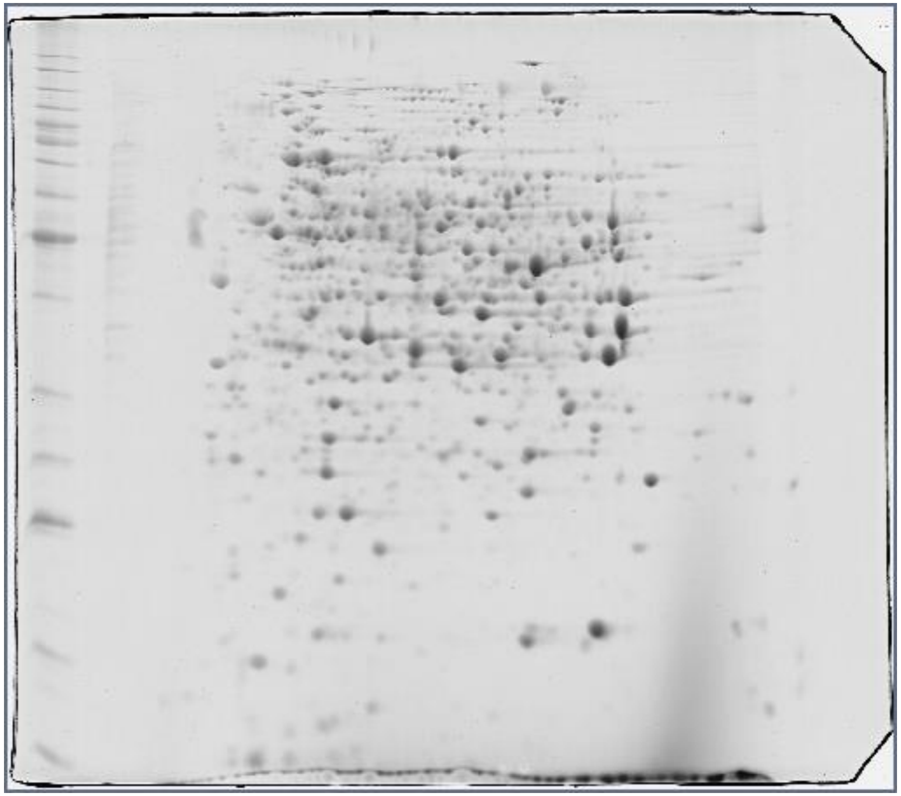

3 stage of TNT transformation, pH 3.6

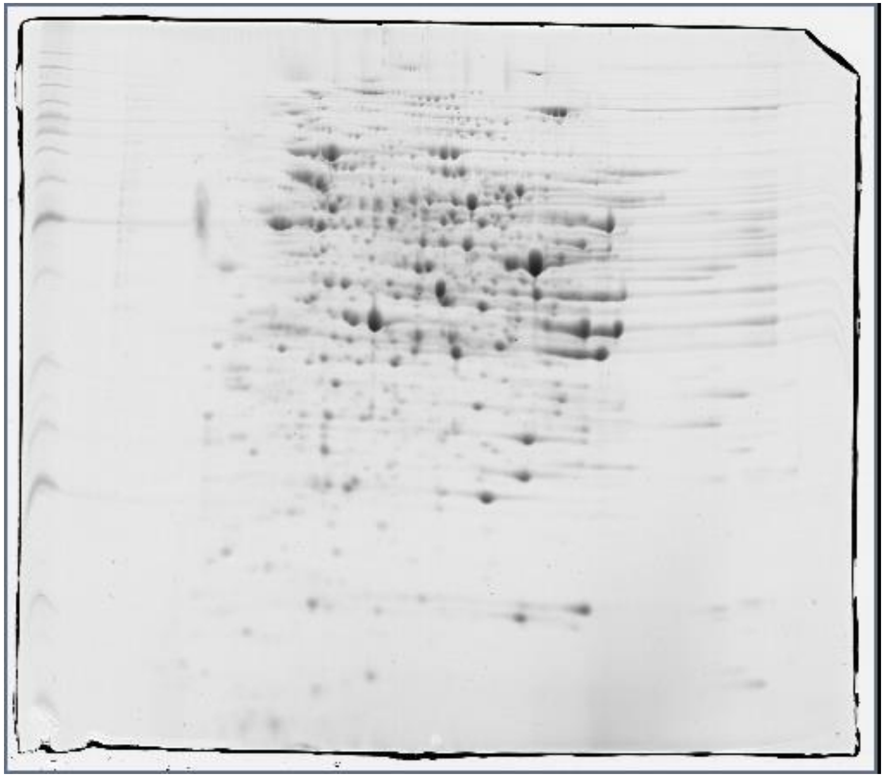

Supplement: Supplementary file 2 [file Data_Sheet_2.PDF]
